# Supplementary material for: Potential and electric double-layer effect in electrocatalytic urea synthesis
Source: Nat Commun. 2024 Feb 6;15:1095. doi: 10.1038/s41467-024-45522-6 (PMC10847171; doi:10.1038/s41467-024-45522-6)
Supplement: Supplementary file 1 — Supplementary Information [file 41467_2024_45522_MOESM1_ESM.pdf]

Potential and electric double-layer effect in electrocatalytic urea synthesis

Qian Wu[1], Chencheng Dai[1,2], Fanxu Meng[1], Yan Jiao[3], Zhichuan J. Xu[1,2,4,5]*

[1] School of Material Science and Engineering, Nanyang Technological University, 50 Nanyang Avenue, Singapore 639798, Singapore

[2] The Cambridge Centre for Advanced Research and Education in Singapore, 1 CREATE way, Singapore 138602, Singapore

[3] School of Chemical Engineering, The University of Adelaide, Adelaide, South Australia 5005, Australia

[4] Energy Research Institute @NTU ERI@N, Interdisciplinary Graduate School, Nanyang Technological University, Singapore 639798, Singapore

[5] Center for Advanced Catalysis Science and Technology, Nanyang Technological University, 50 Nanyang Avenue, Singapore 639798, Singapore

*E-mail: xuzc@ntu.edu.sg

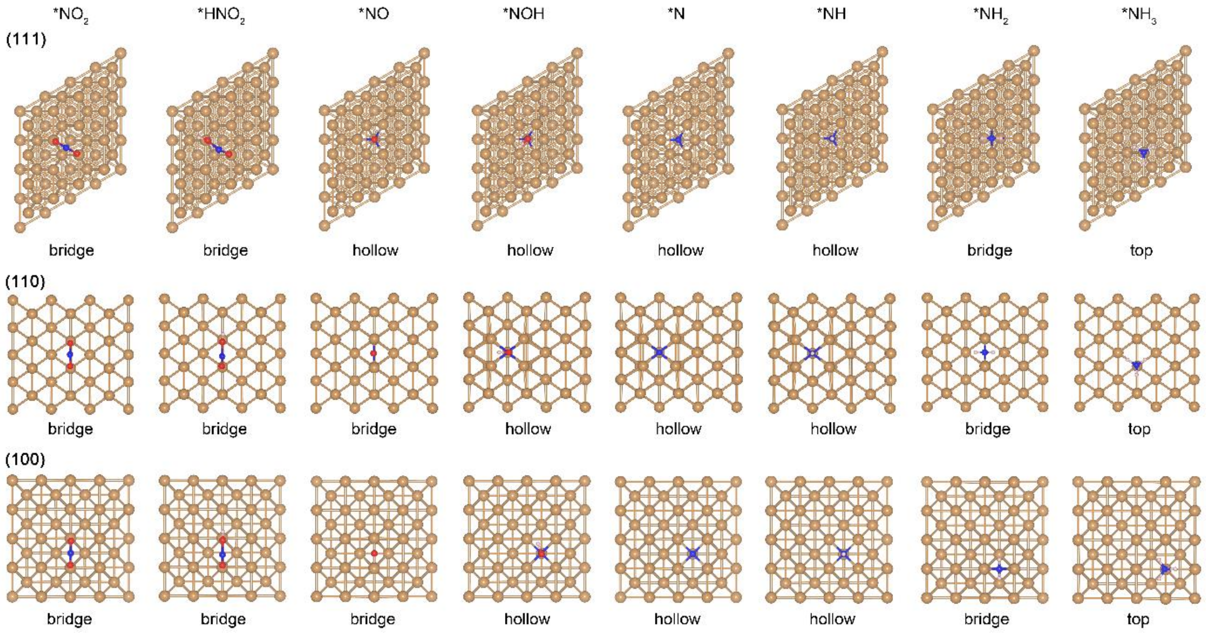

Supplementary Fig. 1 Most stable adsorption configurations and sites of N-intermediates on Cu(111), Cu(110), and Cu(100) surfaces. The brown, blue, pink, and red balls represent copper, nitrogen, hydrogen, and oxygen atoms, respectively.

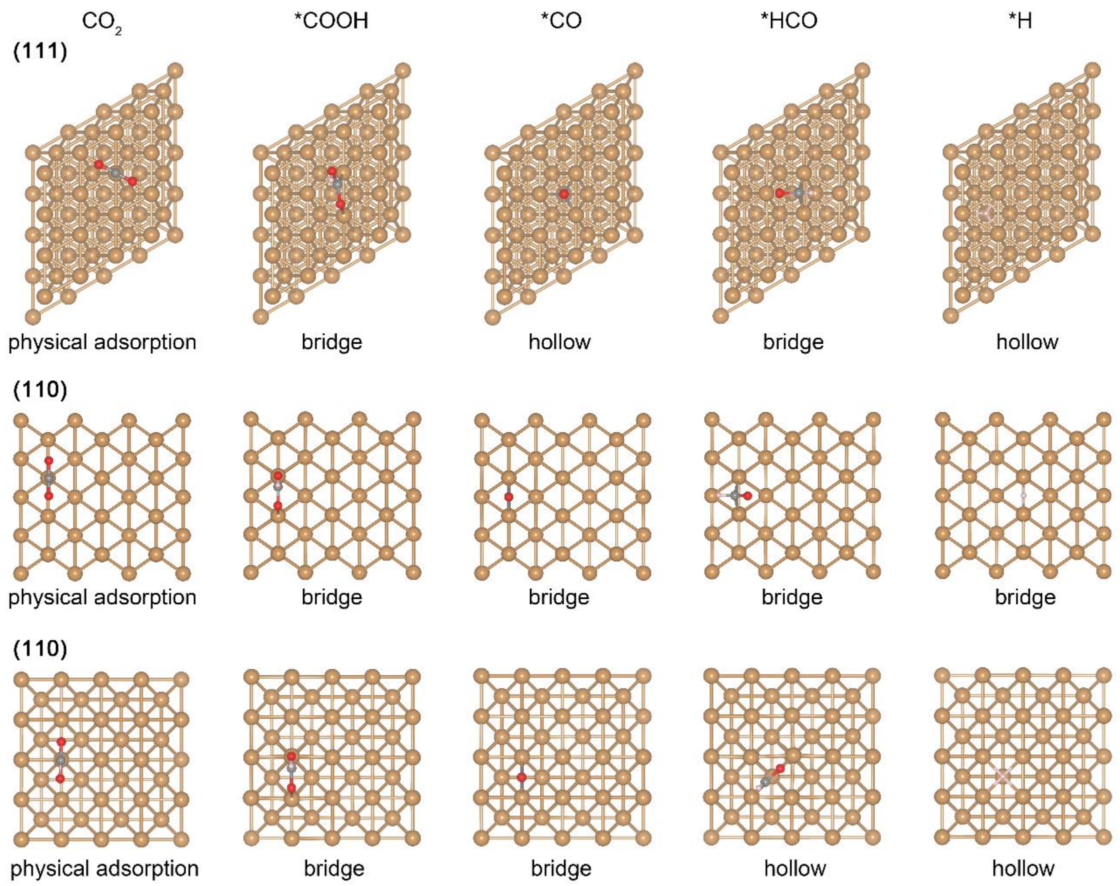

Supplementary Fig. 2 Most stable adsorption configurations and sites of C-intermediates and hydrogen atoms on Cu(111), Cu(110), and Cu(100) surfaces. The brown, grey, pink, and red balls represent copper, carbon, hydrogen, and oxygen atoms, respectively.

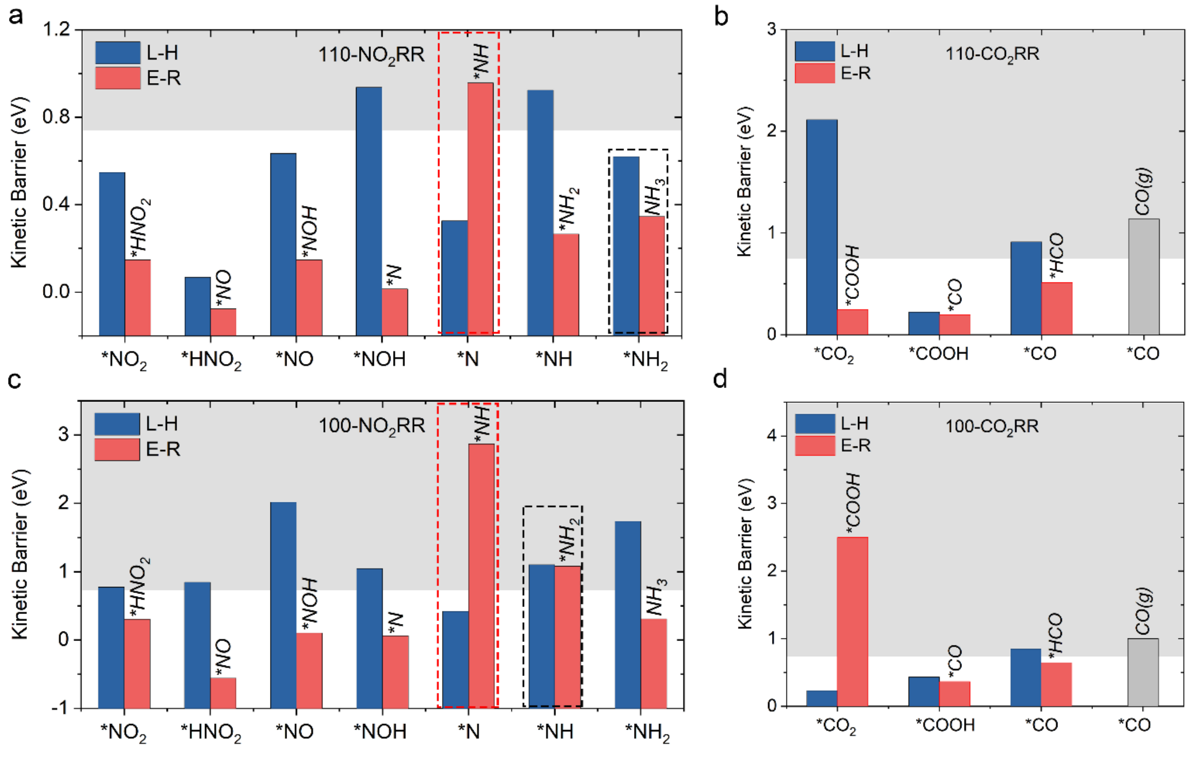

Supplementary Fig. 3 A comparison of the kinetic barrier via L-H and E-R mechanisms for **a,c** NO_2^- RR and **b,d** CO_2RR on Cu(110) and Cu(100) surfaces.

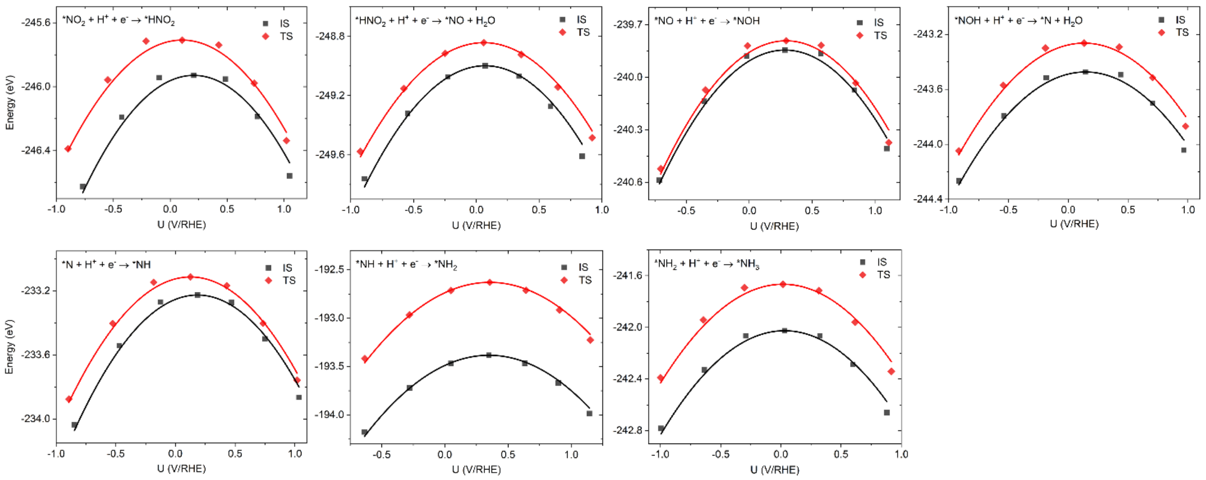

Supplementary Fig. 4 Total energies of the IS and TS via the most favorable mechanism for NO_2^- RR on the charged Cu(111) surface as a quadratic function of U/RHE. The pH is set as 6.8 for NO_2^- RR in accordance with the experimental environments.

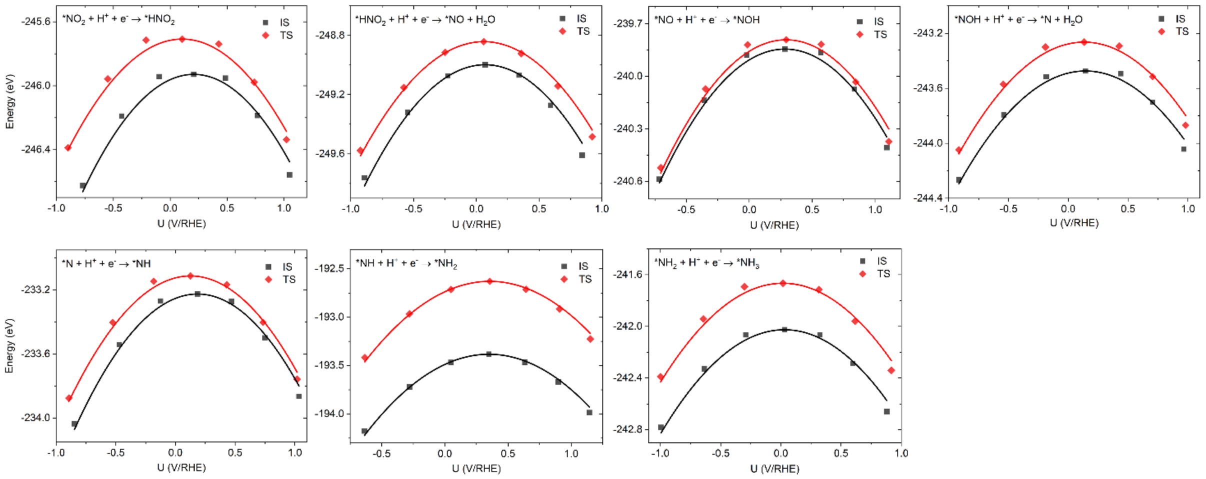

Supplementary Fig. 5 Total energies of the IS and TS via the most favorable mechanism for NO_2^- RR on the charged Cu(110) surface as a quadratic function of U/RHE. The pH is set as 6.8 for NO_2^- RR in accordance with the experimental environments.

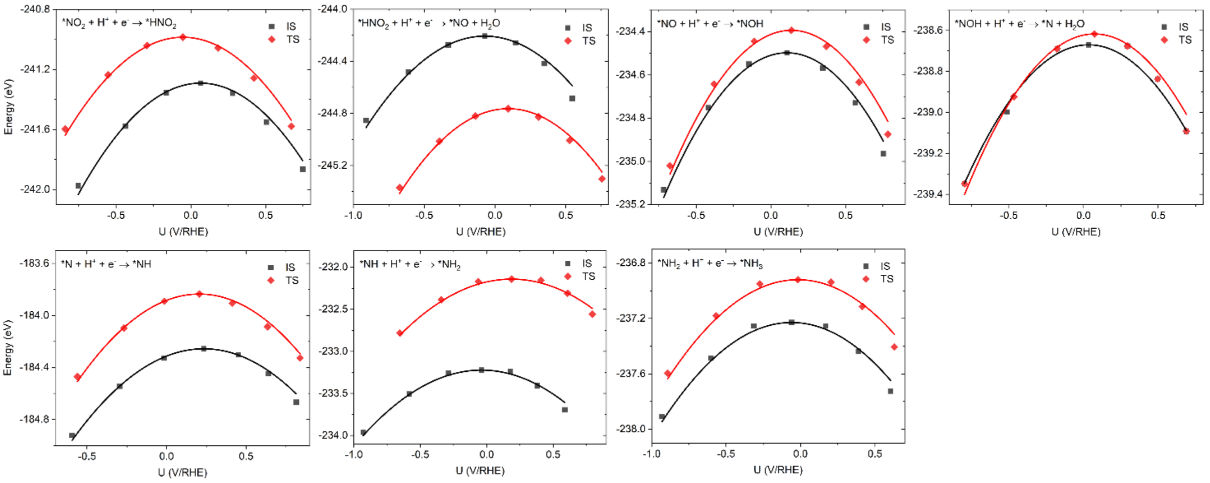

Supplementary Fig. 6 Total energies of the IS and TS via the most favorable mechanism for NO_2^- RR on the charged Cu(100) surface as a quadratic function of U/RHE. The pH is set as 6.8 for NO_2^- RR in accordance with the experimental environments.

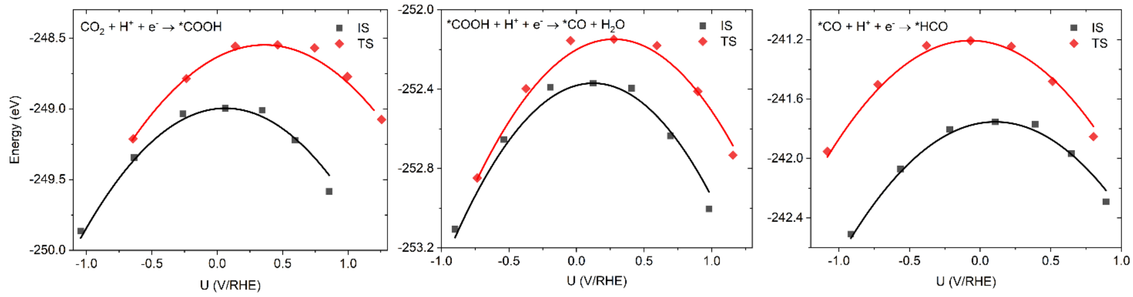

Supplementary Fig. 7 Total energies of the IS and TS via the most favorable mechanism for CO_2RR on the charged Cu(111) surface as a quadratic function of U/RHE. The pH is set as 8.3 for CO_2RR in accordance with the experimental environments.

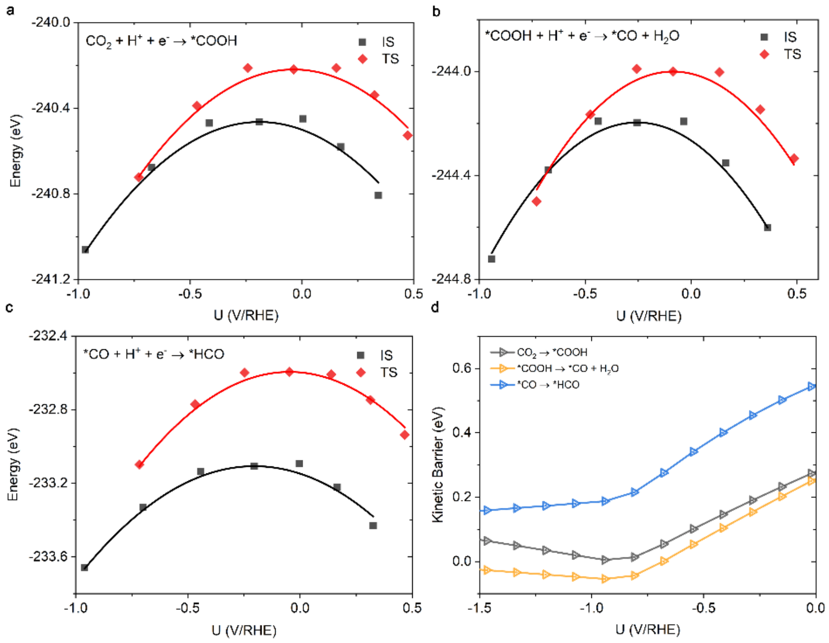

Supplementary Fig. 8 a-c Total energies of the IS and TS via the most favorable mechanism for CO_2RR on the charged Cu(110) surface as a quadratic function of U/RHE. **d** Kinetic barriers CO_2RR hydrogenation steps on the Cu(110) surface hydrogenation as a function of U/RHE (from 0 to -1.5V). The pH is set as 8.3 for CO_2RR in accordance with the experimental environments.

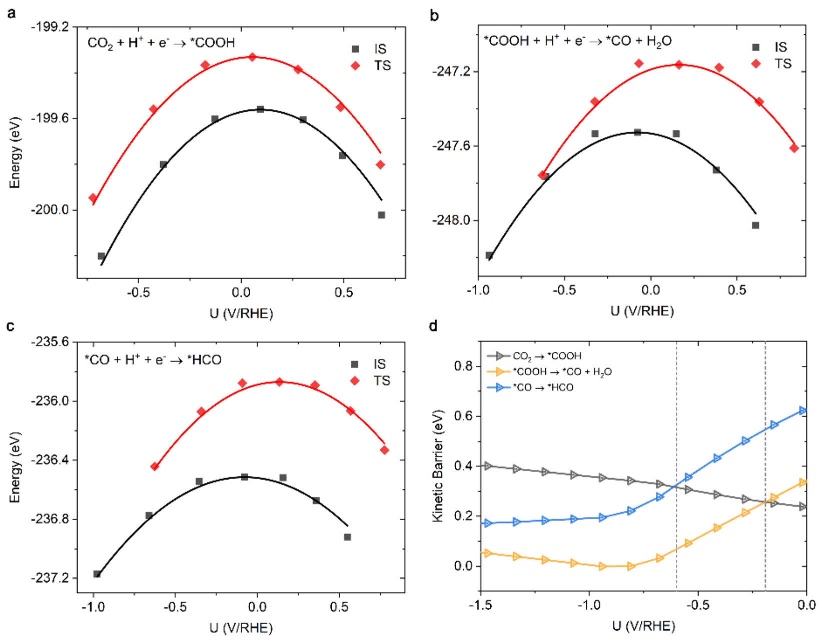

Supplementary Fig. 9 a-c Total energies of the IS and TS via the most favorable mechanism for CO_2RR on the charged Cu(100) surface as a quadratic function of U/RHE. **d** Kinetic barriers for CO_2RR hydrogenation steps on the Cu(100) surface as a function of U/RHE (from 0 to -1.5V). The pH is set as 8.3 for CO_2RR in accordance with the experimental environments.

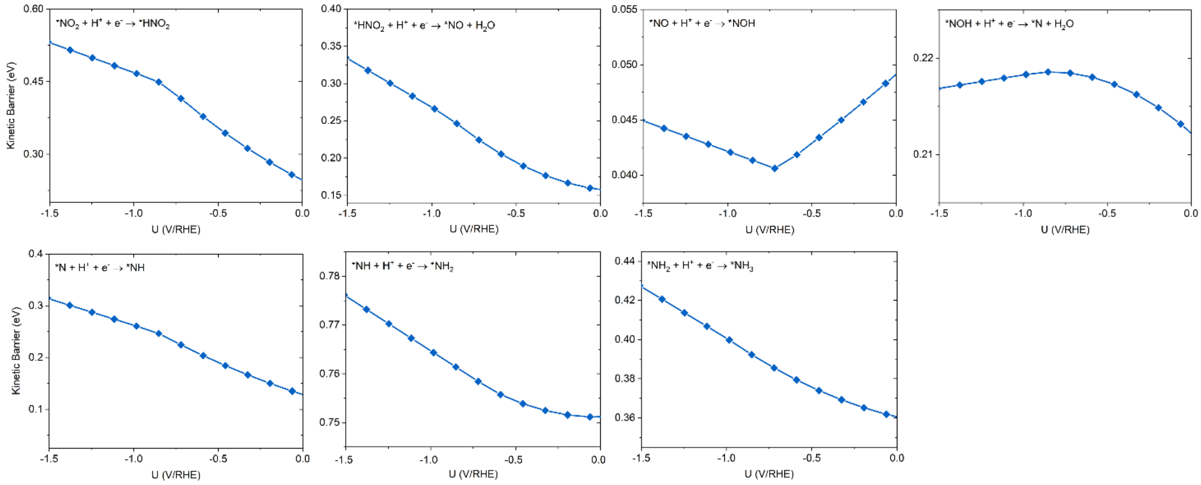

Supplementary Fig. 10 Kinetic barriers for NO_2^-RR hydrogenation steps on the Cu(111) surface as a function of U/RHE (from 0 to -1.5V). The pH is set as 6.8 for NO_2^-RR in accordance with the experimental environments.

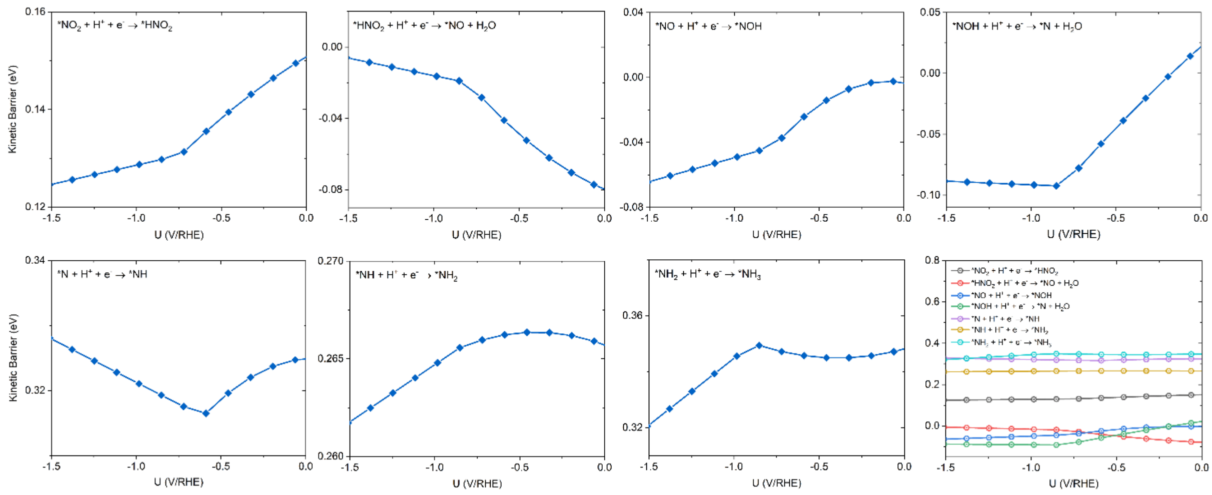

Supplementary Fig. 11 Kinetic barriers for NO_2^-RR hydrogenation steps on the Cu(110) surface as a function of U/RHE (from 0 to -1.5V). The pH is set as 6.8 for NO_2^-RR in accordance with the experimental environments.

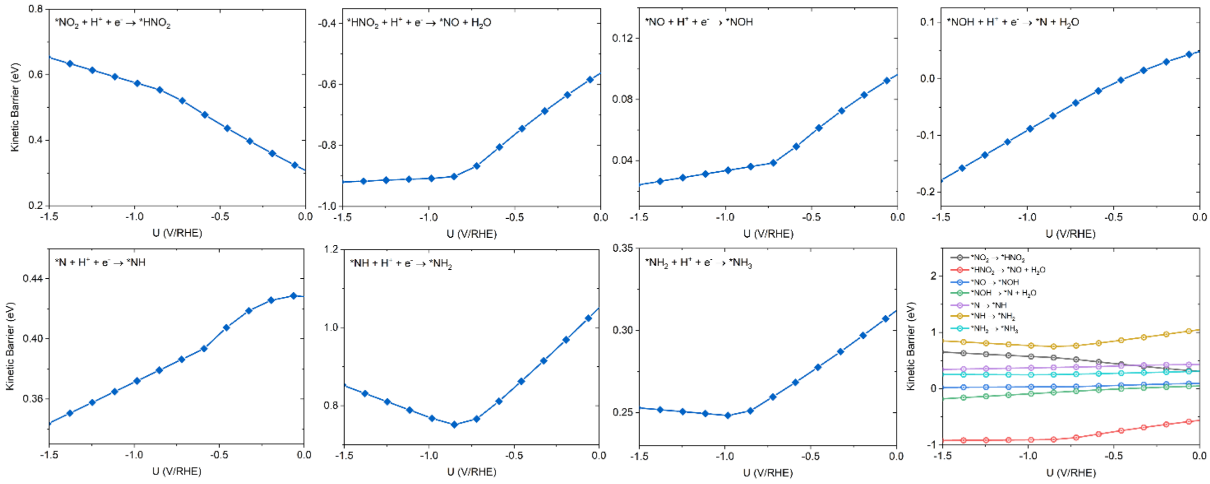

Supplementary Fig. 12 Kinetic barriers for NO_2^-RR hydrogenation steps on the Cu(100) surface as a function of U/RHE (from 0 to -1.5V). The pH is set as 6.8 for NO_2^-RR in accordance with the experimental environments.

Different from the H_3O^+, NO_2^- can not be automatically optimized to the negative charged state due to NO_2 has the other stable neutral state. We add an electron to the systems, enabling to more precisely simulate the interaction between NO_2^- and the charged surface under various electrode potentials. As shown in Supplementary Fig. 13,14, the NO_2^- could maintain the negative charged state under the applied potentials. The results suggest that NO_2^- binds more strongly at more positive potentials.

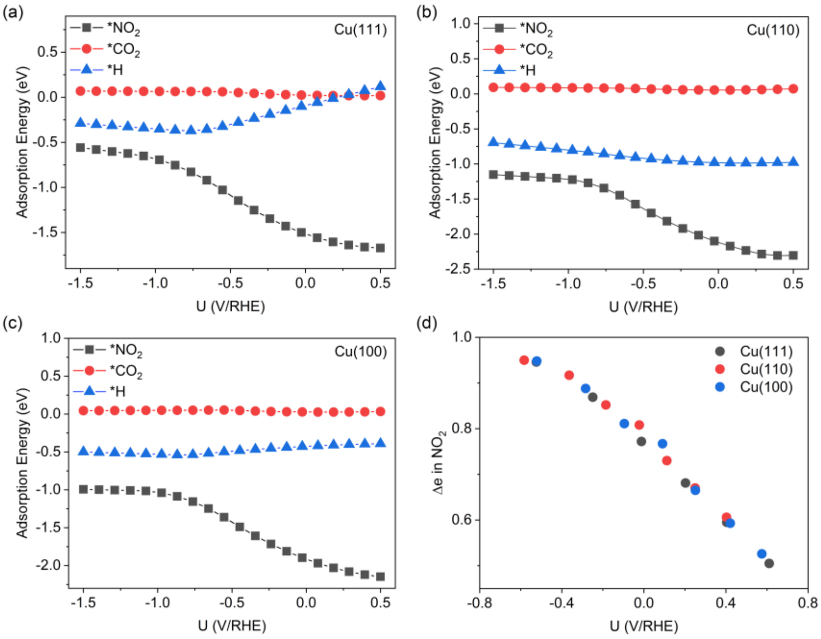

Supplementary Fig. 13 Adsorption energies of $*NO_2$, $*CO_2$, and *H as a function of U/RHE (from 0.50 to -1.50 V) on **a** Cu(111), **b** Cu(110) and **c** Cu(100) surfaces. **d** Excess electrons of NO_2^- in vacuum. The pH is set as 8.3 for urea synthesis in accordance with the experimental environments.

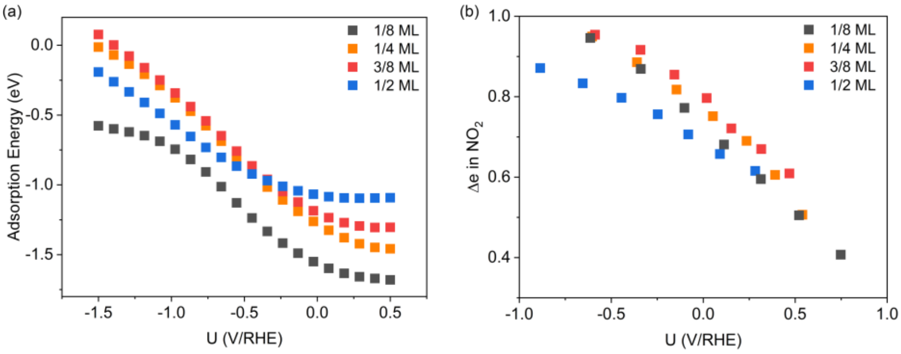

Supplementary Fig. 14 a Adsorption energies of $*NO_2$ at varying coverages as a function of U/RHE (from 0.00 to -1.50 V) on the Cu(111) surface. **b** Excess electrons of NO_2^- in vacuum. The pH is set as 6.8 for NO_2^-RR in accordance with the experimental environments. The ML indicates the monolayer.

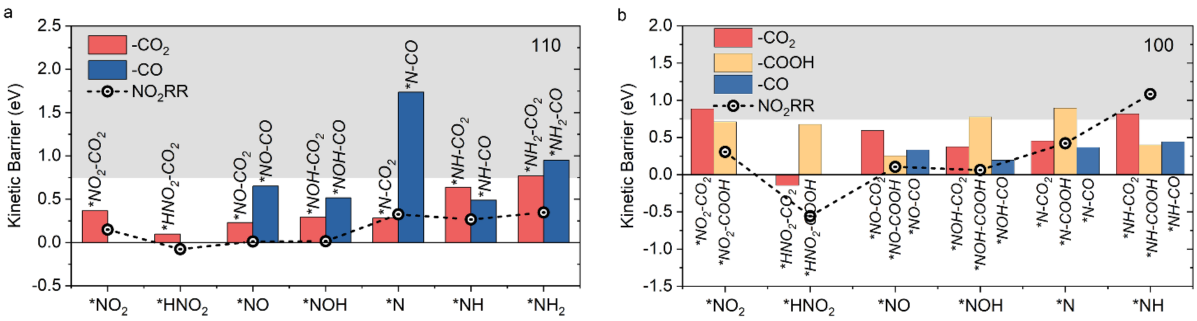

Supplementary Fig. 15 A comparison of the kinetic barrier for the first C-N coupling and NO_2^-RR hydrogenation steps on **a** Cu(111) and **b** Cu(100) surfaces. Blue/Red/yellow bars stand for the kinetic barrier of CO_2/*CO/*COOH coupling steps, the black dashed line stands for the kinetic barrier of NO_2^-RR hydrogenation steps via the favorable mechanism.

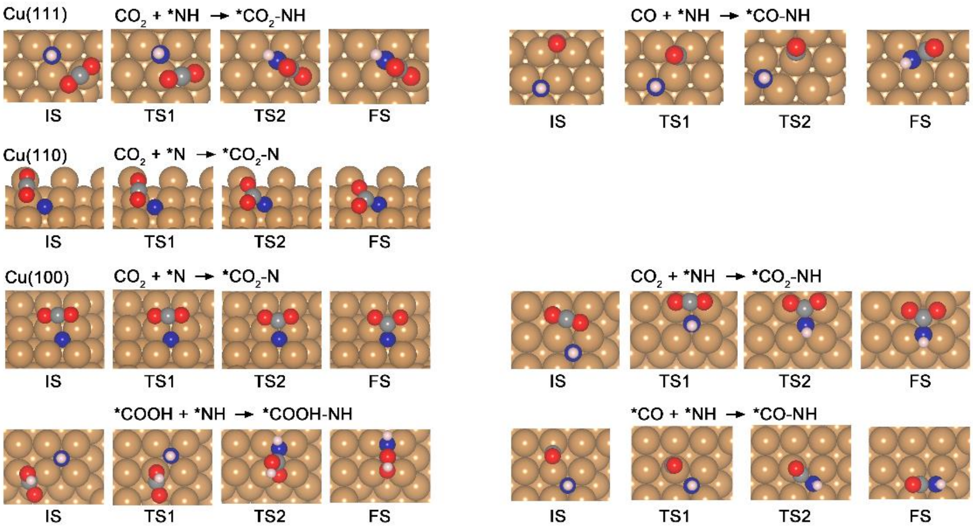

Supplementary Fig. 16 Snapshots of the kinetic process for the first C-N coupling steps on three surfaces.

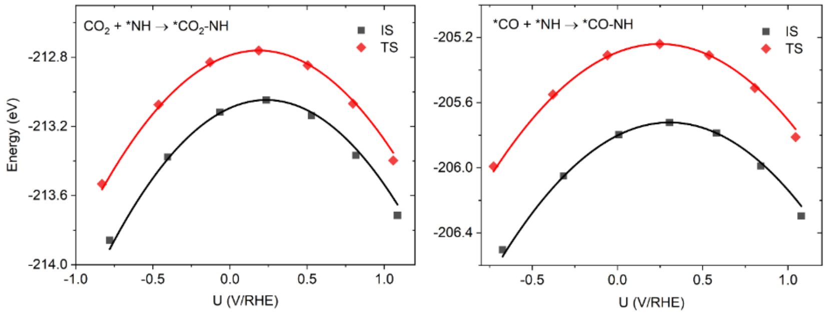

Supplementary Fig. 17 Total energies of the IS and TS for the first C-N coupling step (CO_2 and *NH coupling/*CO and *NH coupling) on the charged Cu(111) surface as a quadratic function of U/RHE. The pH is set as 8.3 for urea synthesis in accordance with the experimental environments.

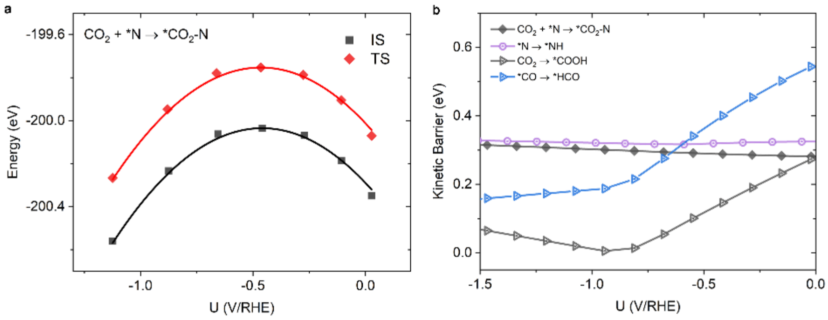

Supplementary Fig. 18 a Total energies of the IS and TS for the first C-N coupling step (CO_2 and *N coupling) on the charged Cu(111) surface as a quadratic function of U/RHE. **b** Kinetic barriers for the first C-N coupling step on the Cu(110) surface as a function of U/RHE (from 0 to -1.5V) and compared to *N, CO_2, and *CO hydrogenation steps. The pH is set as 6.8 for NO_2^-RR and 8.3 for CO_2RR and urea synthesis in accordance with the experimental environments.

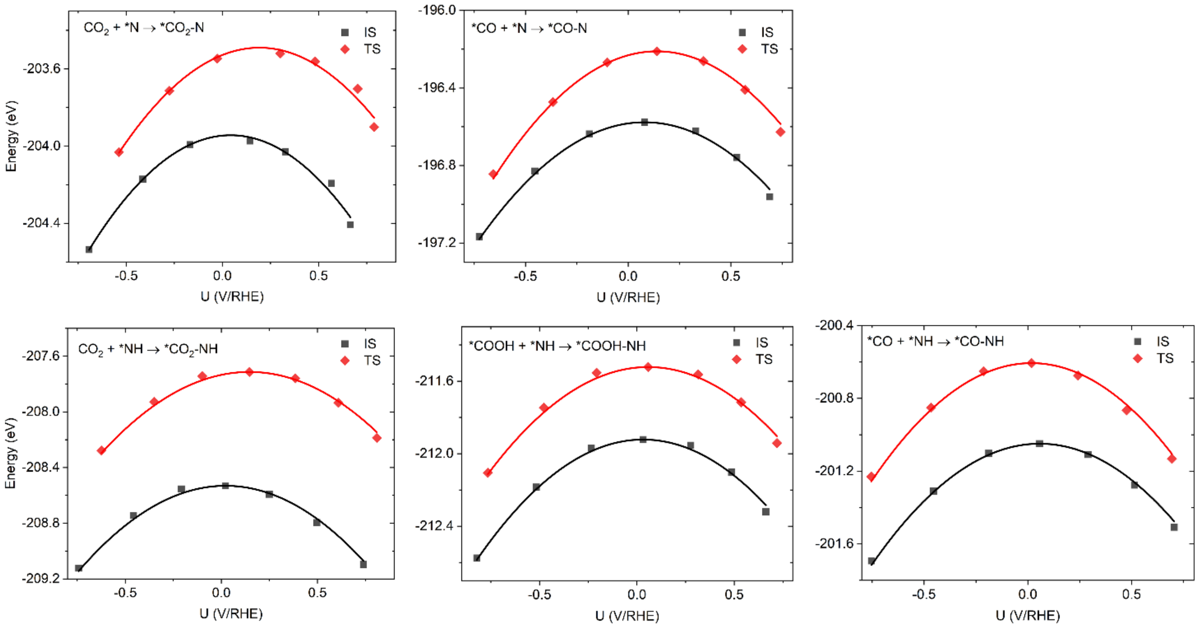

Supplementary Fig. 19 Total energies of the IS and TS for the first C-N coupling step (CO_2 and *N coupling/*CO and *N coupling/CO_2 and *NH coupling/*COOH and *NH coupling/*CO and *NH coupling) on the charged Cu(100) surface as a quadratic function of U/RHE. The pH is set as 8.3 for urea synthesis in accordance with the experimental environments.

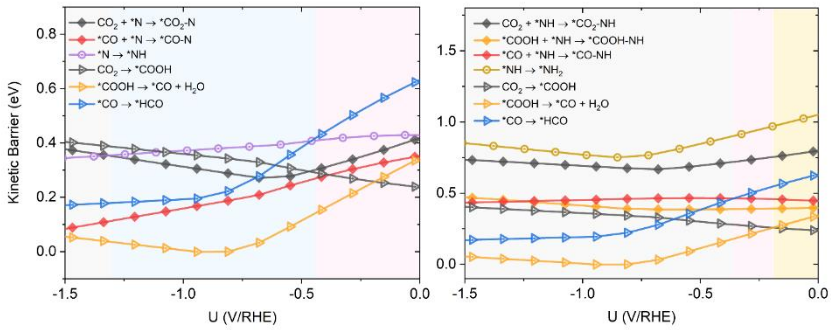

Supplementary Fig. 20 Kinetic barriers for the first C-N coupling step on the Cu(100) surface as a function of U/RHE (from 0 to -1.5V) and compared to the corresponding N- and C-intermediates hydrogenation steps. The pH is set as 6.8 for NO_2^-RR and 8.3 for CO_2RR and urea synthesis in accordance with the experimental environments.

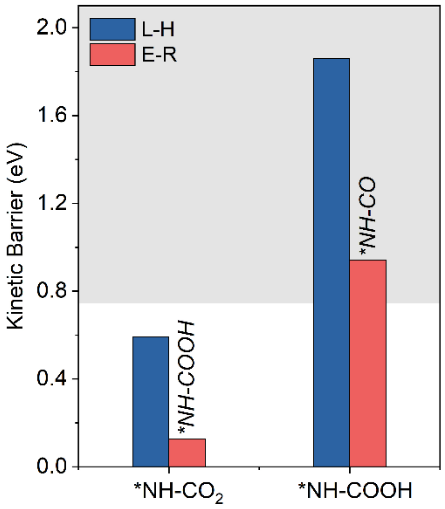

Supplementary Fig. 21 Kinetic barriers for the hydrogenation steps of *CO_2-NH to *COOH-NH and *CO-NH on the Cu(111) surface. The pH is set as 8.3 for urea synthesis in accordance with the experimental environments.

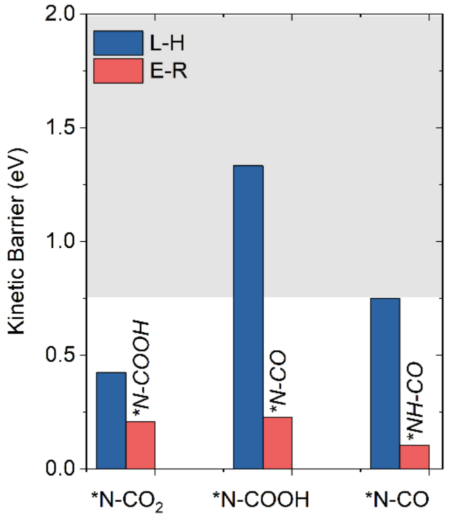

Supplementary Fig. 22 Kinetic barriers for the hydrogenation steps of *CO_2-N to *COOH-N, *CO-N, and *CO-NH on the Cu(100) surface. The pH is set as 8.3 for urea synthesis in accordance with the experimental environments.

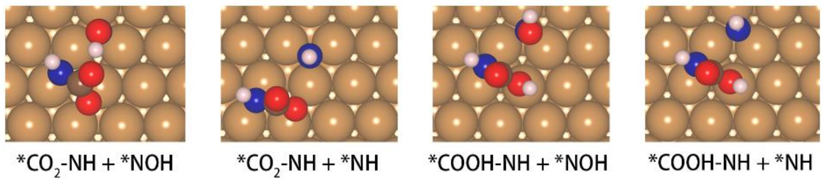

Supplementary Fig. 23 Optimized structure configurations of the second C-N coupling on the Cu(111) surface for *CO_2-NH + *NOH, *CO_2-NH + *NH, *COOH-NH + *NOH and *COOH-NH + *NH.

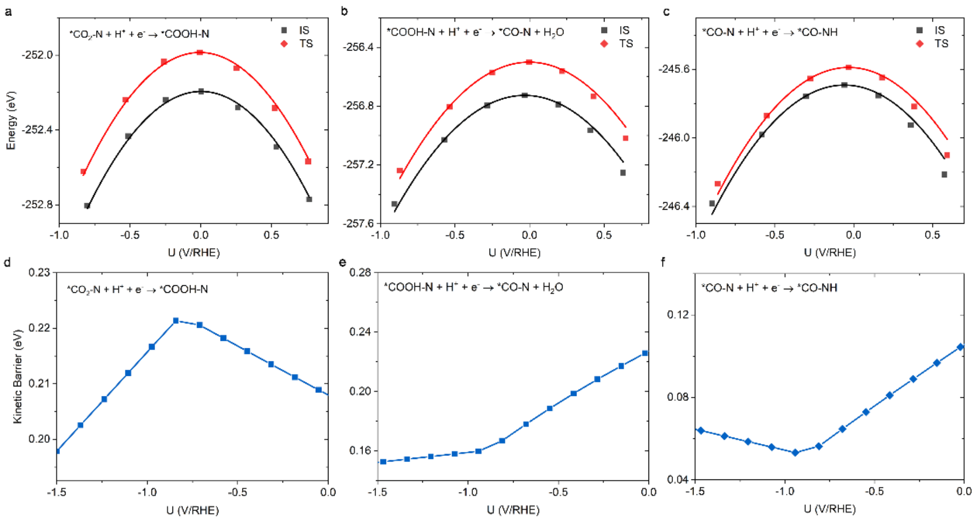

Supplementary Fig. 24 a-c Total energies of the IS and TS for the hydrogenation steps of *CO_2-N to *COOH-N, *CO-N, and *CO-NH on the charged Cu(100) surface as a quadratic function of U/RHE. **d-f** Kinetic barriers for the hydrogenation steps of *CO_2-N to *COOH-N, *CO-N, and *CO-NH on the Cu(110) surface as a function of U/RHE (0 to -1.5V). The pH is set as 8.3 for urea synthesis

in accordance with the experimental environments.

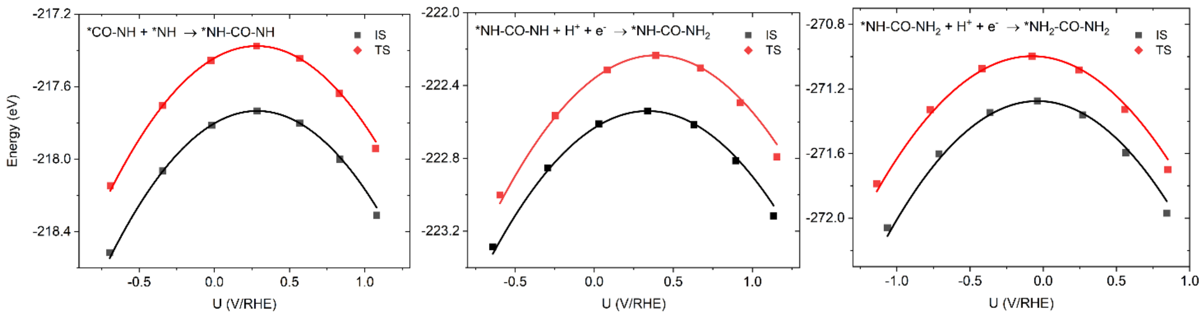

Supplementary Fig. 25 Total energies of the IS and TS for the second C-N coupling step (*CO-NH and *NH coupling) and the further hydrogenation steps for *NH-CO-NH to *NH-CO-NH$_2$ and CO(NH$_2$)$_2$ on the charged Cu(111) surface as a quadratic function of U/RHE. The pH is set as 8.3 for urea synthesis in accordance with the experimental environments.

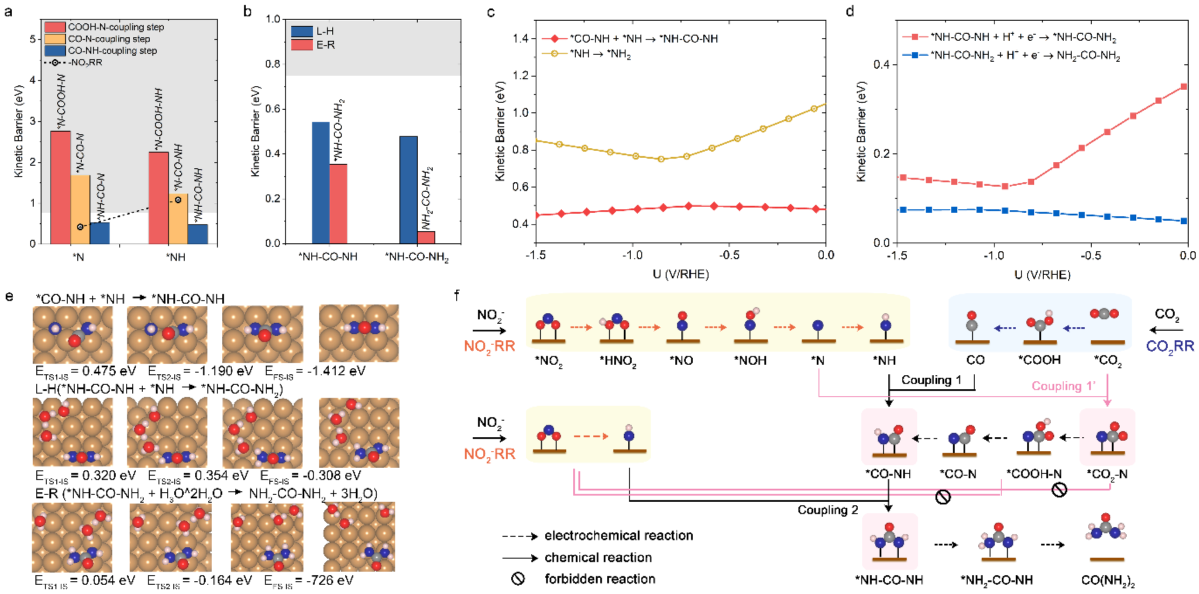

Supplementary Fig. 26 a A comparison of the kinetic barrier for the second C-N coupling and the corresponding NO_2^-RR hydrogenation steps on the Cu(100) surface. **b** A comparison of the kinetic barrier via L-H and E-R mechanisms for the hydrogenation step of *NH-CO-NH and *NH-CO-NH$_2$ on the Cu(100) surface. **c** Kinetic barriers for *CO-NH and *NH coupling step as a function of the U/RHE, compared to the *NH hydrogenation step. **d** Kinetic barriers for *NH-CO-NH and *NH-CO-NH$_2$ hydrogenation steps as a function of the U/RHE. **e** Snapshots of the kinetic process for the *CO-NH and *NH coupling and *NH-CO-NH and *NH-CO-NH$_2$ hydrogenation steps. **f** Schematic illustration of urea synthesis on the Cu(100) surface.

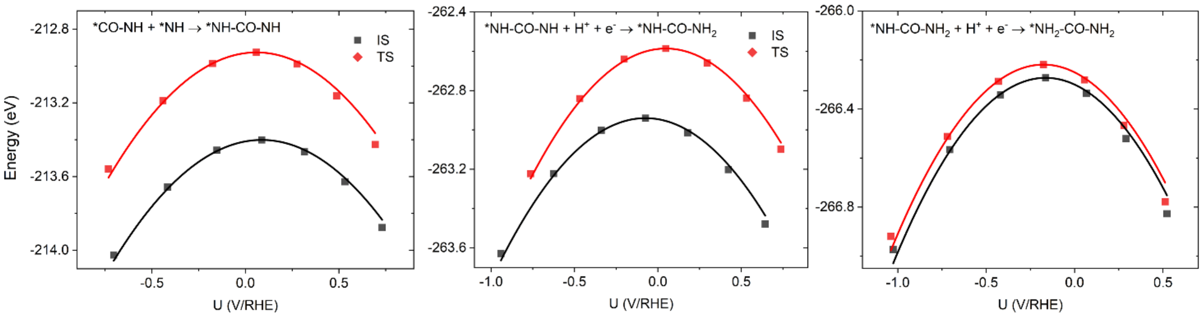

Supplementary Fig. 27 Total energies of the IS and TS for the second C-N coupling step (*CO-NH and *NH coupling) and the further hydrogenation steps for *NH-CO-NH to *NH-CO-NH_2 and $CO(NH_2)_2$ on the charged Cu(100) surface as a quadratic function of U/RHE. The pH is set as 8.3 for urea synthesis in accordance with the experimental environments.

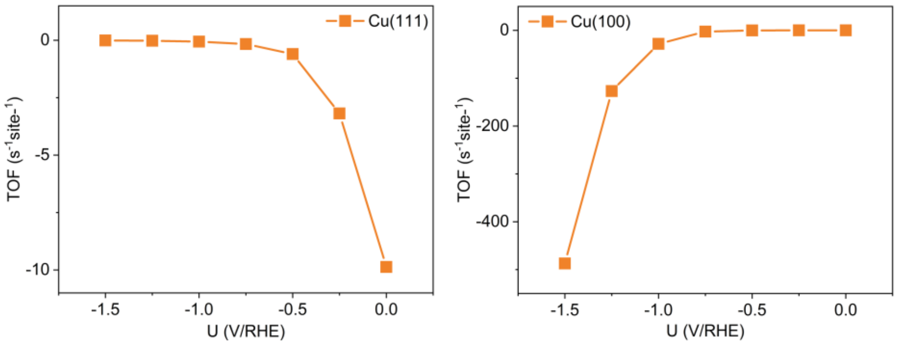

Supplementary Fig. 28 TOF for CO_2RR to CO on Cu(111) and Cu(100) surfaces as functions of applied electrode potential vs RHE at 300 K and 1 bar. The pH is set as 8.3 for CO_2RR in accordance with the experimental environments.

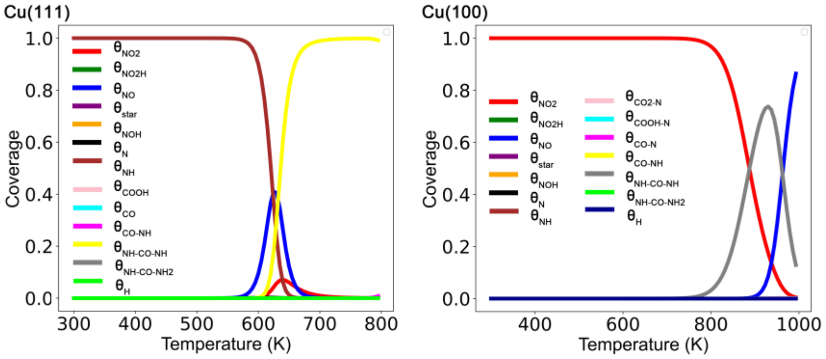

Supplementary Fig. 29 Simulated coverage curves of adsorbed species for urea synthesis as a function of temperature on Cu(111) (300 to 800K) and Cu(100) (300 to 1000K) under the applied electrode potential of -1.50 and -0.50 V, respectively. The pH is set as 8.3 for urea synthesis in accordance with the experimental environments.

To deepen our understanding of the mechanism, ab initio molecular dynamics (AIMD) simulations are conducted for *NO_2 adsorbed Cu(111) and Cu(100). Supplementary Fig. 30 indicates that *NO_2 maintains stable adsorption on the Cu(111) surface at 300 and 500 K. However, at higher temperatures (700 and 900 K), *NO_2 dissociates into *NO and *O on the Cu(111) surface, while it remains stable across the simulated temperature range (300 to 900 K) on the Cu(100) surface.

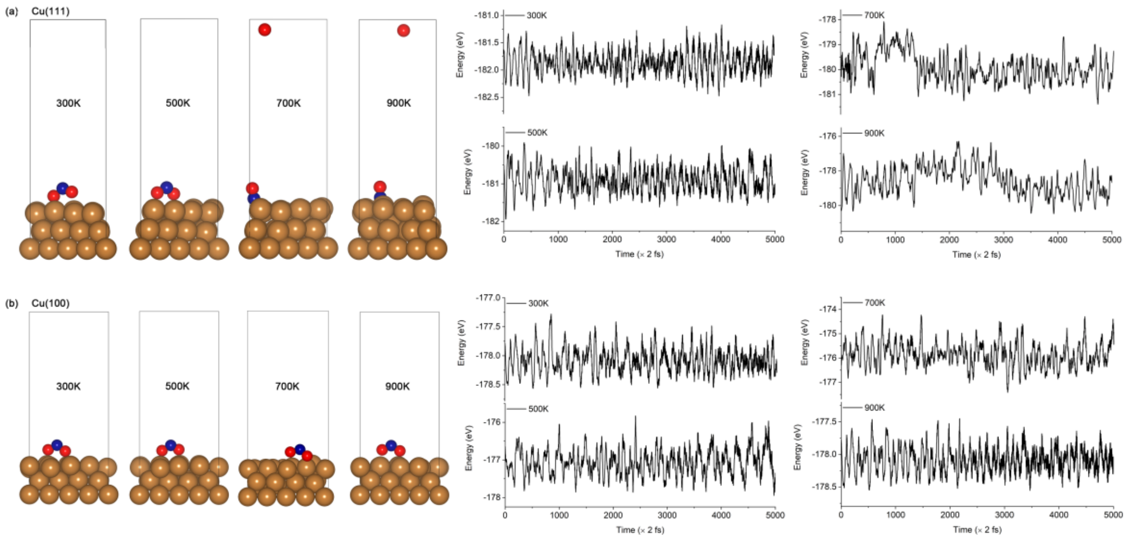

Supplementary Fig. 30 Snapshot of atomic configuration (left panels) and the corresponding variations of energy against the time (right panels) for AIMD simulation of *NO_2 adsorbed **a** Cu(111) and **b** Cu(100) at 300/500/700/900 K for 10 ps with a time step of 2 fs.

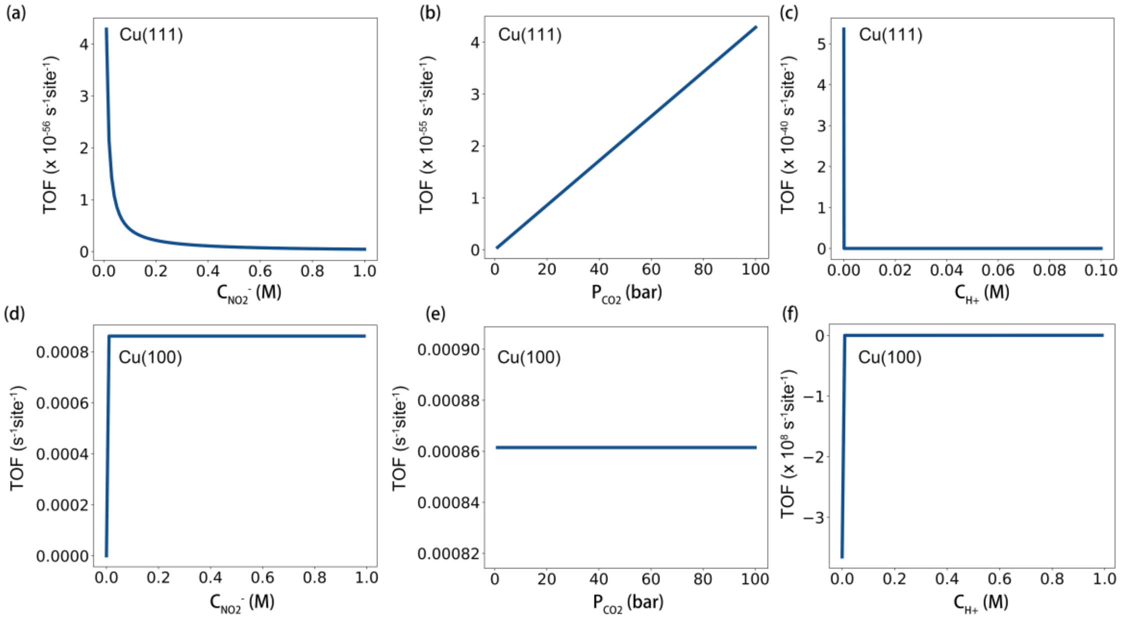

Supplementary Fig. 31 TOF of urea synthesis as a function of **a** NO_2^- concentration (0 to 1 M), **b** CO_2 pressure (0 to 100 bar) and **c** H^+ concentration (0 to 0.1 M) on Cu(111) surface under the temperature of 300 K and the applied potential of -1.50. TOF of urea synthesis as a function of **d** NO_2^- concentration (0 to 1 M), **e** CO_2 pressure (0 to 100 bar) and **f** H^+ concentration (0 to 0.1 M) on Cu(100) surface under the temperature of 300 K and the applied potential of -0.50 V,

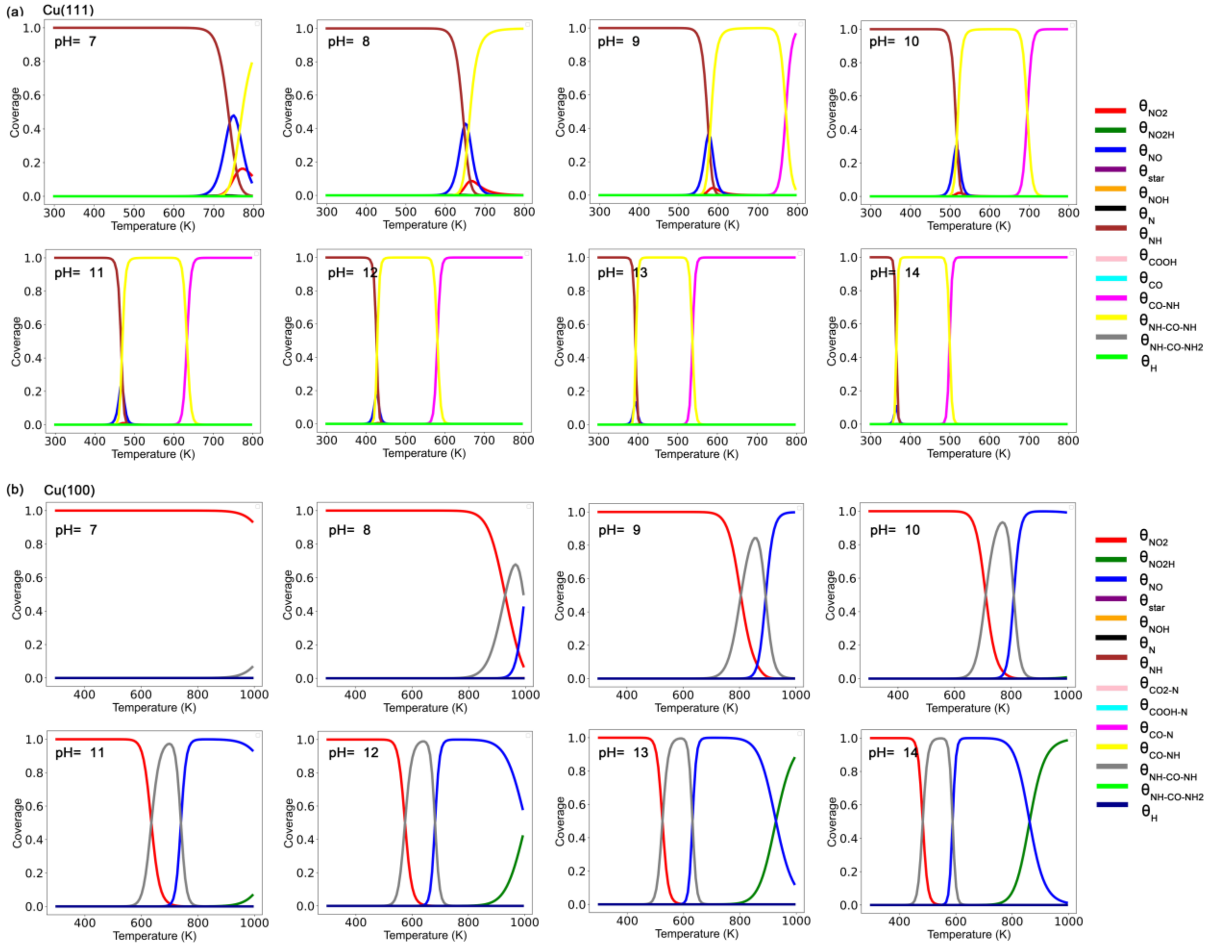

Supplementary Fig. 32 Simulated coverage curves of adsorbed species for urea synthesis as a function of temperature on **a** Cu(111) and **b** Cu(100) under different pH values (pH = 7 to 14). The applied potential of -1.50 and -0.50 V, respectively.

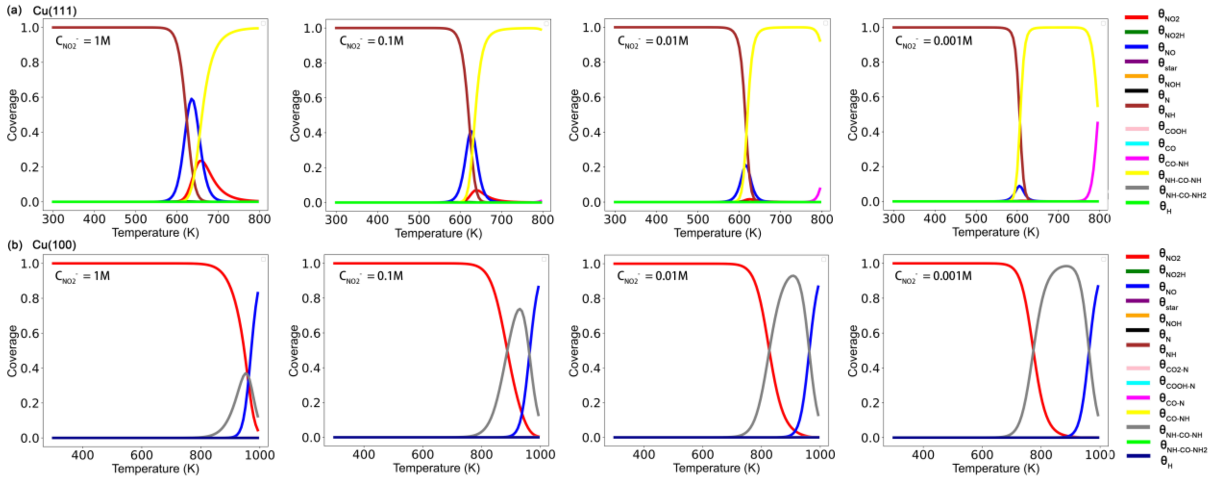

Supplementary Fig. 33 Simulated coverage curves of adsorbed species for urea synthesis as a function of temperature on **a** Cu(111) and **b** Cu(100) surfaces under different NO^- concentrations (C_{NO2-} = 0.001 to 1 M). The applied potential of -1.50 and -0.50 V, respectively. The pH is set as 8.3 for urea synthesis in accordance with the experimental environments.

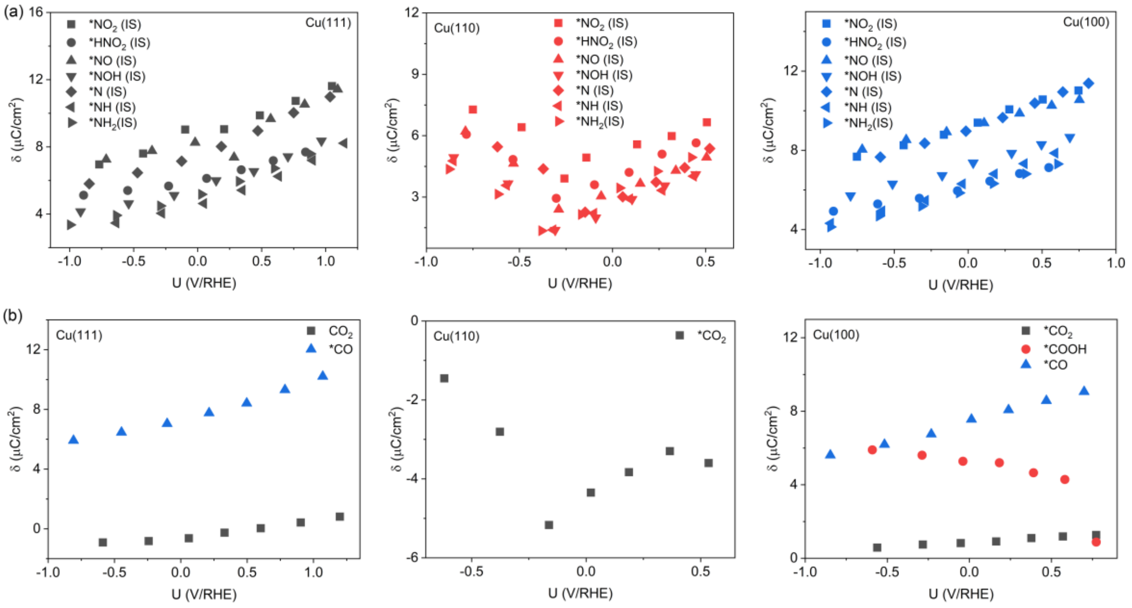

Supplementary Fig. 34 Change in surface-charge density (σ) for **a** N-intermediates or **b** C-intermediates adsorbed Cu(111), Cu(110) and Cu(100) surfaces as a function of applied electrode potential vs RHE. The surface charge is defined as the number of excess electrons per unit area.[1]

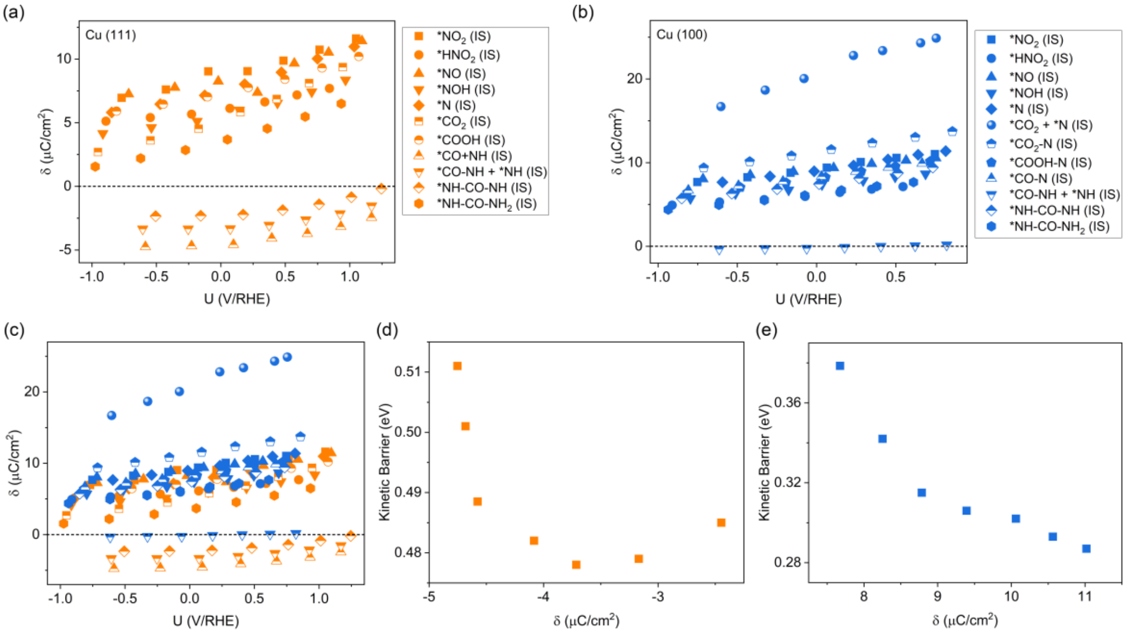

Supplementary Fig. 35 Change in surface-charge density (σ) for intermediates adsorbed **a** Cu(111) and **b** Cu(100) surfaces as a function of applied electrode potential vs RHE during the urea synthesis process. **c** Comparison the surface-charge density of Cu(111) and Cu(100) surfaces during the urea synthesis. Kinetic barriers for the RDS of **d** Cu(111) and **e** Cu(100) during the urea synthesis process as a function of the surface-charge density. The surface charge density is defined as the number of excess electrons per unit area.

Taken NO_2^-RR of Cu(111), Cu(110), and Cu(100) surfaces as examples. Bader charge analysis is employed to investigate the electron distribution of H_3O^+ during the proton-coupled electron transfer (PECT) steps. As demonstrated in Supplementary Fig. 36, H_3O^+ loses more than 0.5 electrons under the applied electrode potential, thereby existing in an ionic state.

The ions in the bulk electrolyte using DFT is challenging due to limitations in unit cell size and the

use of static solvation models. In light of this, we further compare our method with another approximating method for electrode potential-dependent activation energies proposed by Akhade, S. A., et al.[2-5] Supplementary Fig. 37 shows the optimized structures of the reactant (*NO_2 + 3H_2O), reference (*NO_2 + 3H_2O + *H), transition (*NO_2 + 2H_2O + H_3O^+), and product (*HNO_2 + 3H_2O) states used to calculate the potential-dependent kinetic barrier for hydrogenation step of *NO_2 on Cu(111) surface. The kinetic barrier is 0.402 eV at U_0 = 0.37 V vs RHE with pH of 8.3 (the experimental conditions for urea synthesis).

The kinetic barrier can be approximated as a linear function of the electrode potential using Bulter-Volmer theory:[2]

$$\Delta G_{act} = \Delta G_{act(U_0)} + \beta F(U\text{-}U_0)$$

where F is the Faraday's constant, $F(U - U_0)$ is the kinetic barrier change due to the altered electrode potential and β is the reaction symmetry factor, approximated as 0.5 based on previous studies.[2] As depicted in Supplementary Fig. 38, the kinetic barrier calculated using the Butler-Volmer theory is lower than our initial results. This further substantiates our conclusion that Eley-Rideal (E-R) mechanisms are more favorable than Langmuir-Hinshelwood (L-H) mechanisms. Therefore, despite representing ions in the bulk electrolyte using DFT is challenging due to limitations in unit cell size and the use of static solvation models, our results still provide valuable insights and offer a reasonable approximation.

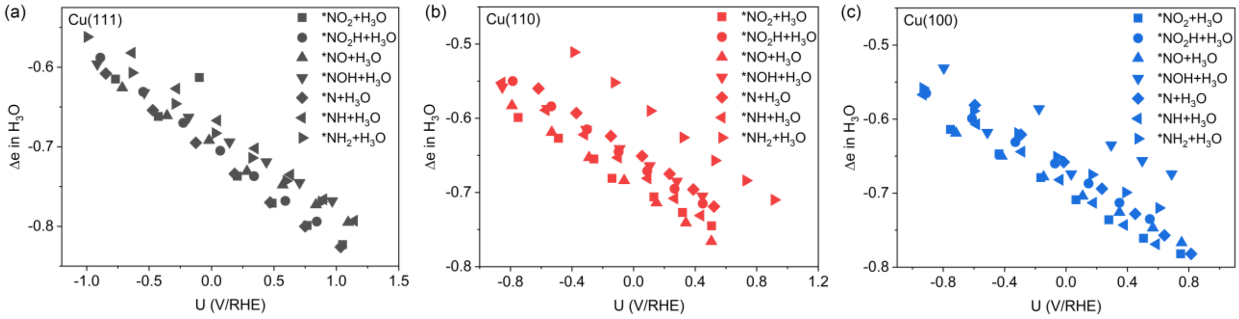

Supplementary Fig. 36 Excess electrons of H_3O^+ in vacuum during the NO_2^-RR on **a** Cu(111), **b** Cu(110) and **c** Cu(100) surfaces.

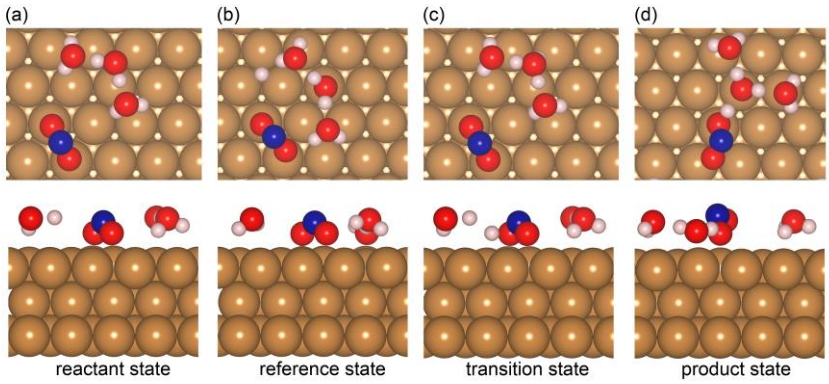

Supplementary Fig. 37 Top and side view of the **a** reactant, **b** reference, **c** transition and **d** product state for the hydrogenation step of *NO_2 to *HNO_2 on the Cu (111) surface via E-R mechanism.

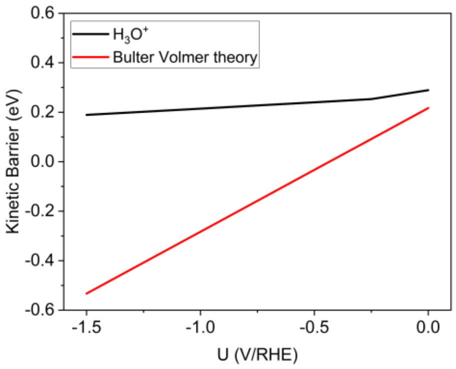

Supplementary Fig. 38 Comparison of the potential-dependent kinetic barrier (eV) for the hydrogenation step of *NO_2 on the Cu (111) surface via the method of this work and Bulter Volmer theory with β equal to 0.5.

Supplementary Table 1 Fitted parameters for potential-dependent total energies of initial and transition state during the NO_2^-RR on the Cu(111) surface.

	C (μF/cm^2)	U_0 (V)	E_0 (eV)	R^2
*NO_2 + H^+ + e^- (IS)	27.152	-0.283	-245.929	0.970
*NO_2 + H^+ + e^- (TS)	24.228	-0.385	-245.708	0.979
*HNO_2 + H^+ + e^- (IS)	31.258	-0.420	-248.998	0.978
*HNO_2 + H^+ + e^- (TS)	28.192	-0.431	-248.842	0.992
*NO + H^+ + e^- (IS)	26.976	-0.206	-239.844	0.984
*NO + H^+ + e^- (TS)	26.941	-0.197	-239.791	0.979
*NOH + H^+ + e^- (IS)	25.760	-0.346	-243.474	0.982
*NOH + H^+ + e^- (TS)	26.095	-0.358	-243.264	0.982
*N + H^+ + e^- (IS)	28.016	-0.305	-233.225	0.987
*N + H^+ + e^- (TS)	26.606	-0.364	-233.112	0.990
*NH + H^+ + e^- (IS)	30.412	-0.141	-193.383	0.988
*NH + H^+ + e^- (TS)	29.919	-0.135	-192.630	0.988
*NH_2 + H^+ + e^- (IS)	26.765	-0.460	-242.026	0.977
*NH_2 + H^+ + e^- (TS)	26.06	-0.473	-241.666	0.976
*NH_2 + H^+ + e^- (TS)	26.06	-0.473	-241.666	0.976

Supplementary Table 2 Fitted parameters for potential-dependent total energies of initial and transition state during the NO_2^-RR on the Cu(110) surface.

	C (μF/cm^2)	U_0 (V)	E_0 (eV)	R^2
*NO_2 + H^+ + e^- (IS)	37.443	-0.560	-237.982	0.995
*NO_2 + H^+ + e^- (TS)	37.803	-0.560	-237.834	0.997
*HNO_2 + H^+ + e^- (IS)	36.407	-0.587	-240.861	0.982
*HNO_2 + H^+ + e^- (TS)	35.184	-0.607	-240.936	0.976
*NO + H^+ + e^- (IS)	32.003	-0.551	-230.932	0.990
*NO + H^+ + e^- (TS)	34.536	-0.554	-230.933	0.995
*NOH + H^+ + e^- (IS)	34.435	-0.580	-234.449	0.981
*NOH + H^+ + e^- (TS)	35.068	-0.528	-234.435	0.984
*N + H^+ + e^- (IS)	36.061	-0.435	-180.512	0.986
*N + H^+ + e^- (TS)	36.651	-0.435	-180.187	0.994
*NH + H^+ + e^- (IS)	34.464	-0.590	-229.184	0.980
*NH + H^+ + e^- (TS)	34.579	-0.591	-228.918	0.983
*NH_2 + H^+ + e^- (IS)	34.306	-0.658	-233.920	0.995
*NH_2 + H^+ + e^- (TS)	33.701	-0.654	-233.574	0.977
*NH_2 + H^+ + e^- (TS)	37.443	-0.560	-237.982	0.995

Supplementary Table 3 Fitted parameters for potential-dependent total energies of initial and transition state during the NO_2^-RR on the Cu(100) surface.

	C (μF/cm^2)	U_0 (V)	E_0 (eV)	R^2
*NO_2 + H^+ + e^- (IS)	33.790	-0.425	-241.291	0.978
*NO_2 + H^+ + e^- (TS)	32.133	-0.543	-240.985	0.987
*HNO_2 + H^+ + e^- (IS)	30.354	-0.562	-244.207	0.959
*HNO_2 + H^+ + e^- (TS)	33.668	-0.397	-244.764	0.982
*NO + H^+ + e^- (IS)	29.868	-0.382	-234.498	0.982
*NO + H^+ + e^- (TS)	30.689	-0.353	-234.394	0.978
*NOH + H^+ + e^- (IS)	29.777	-0.456	-238.670	0.982
*NOH + H^+ + e^- (TS)	31.525	-0.417	-238.617	0.974
*N + H^+ + e^- (IS)	31.221	-0.256	-184.256	0.982
*N + H^+ + e^- (TS)	34.854	-0.284	-183.835	0.987
*NH + H^+ + e^- (IS)	29.807	-0.529	-233.223	0.976
*NH + H^+ + e^- (TS)	28.637	-0.303	-232.141	0.978
*NH_2 + H^+ + e^- (IS)	28.743	-0.550	-237.229	0.975
*NH_2 + H^+ + e^- (TS)	28.454	-0.508	-236.920	0.969
*NH_2 + H^+ + e^- (TS)	33.790	-0.425	-241.291	0.978

Supplementary Table 4 Fitted parameters for potential-dependent total energies of initial and transition state during the CO_2RR on the Cu(111) surface.

	C (μF/cm^2)	U_0 (V)	E_0 (eV)	R^2
CO_2 + H^+ + e^- (IS)	26.465	-0.338	-248.996	0.970
CO_2 + H^+ + e^- (TS)	23.241	-0.039	-248.548	0.969
*COOH + H^+ + e^- (IS)	24.351	-0.277	-252.370	0.972
*COOH + H^+ + e^- (TS)	26.254	-0.124	-252.148	0.980
*CO + H^+ + e^- (IS)	26.395	-0.293	-241.754	0.981
*CO + H^+ + e^- (TS)	26.835	-0.468	-241.207	0.983

Supplementary Table 5 Fitted parameters for potential-dependent total energies of initial and transition state during the CO_2RR on the Cu(110) surface.

	C (μF/cm^2)	U_0 (V)	E_0 (eV)	R^2

$CO_2 + H^+ + e^-$ (IS)	28.823	-0.590	-240.464	0.970
$CO_2 + H^+ + e^-$ (TS)	30.032	-0.437	-240.219	0.969
*COOH + $H^+ + e^-$ (IS)	26.722	-0.477	-247.527	0.978
*COOH + $H^+ + e^-$ (TS)	27.240	-0.237	-247.163	0.981
*CO + $H^+ + e^-$ (IS)	28.017	-0.604	-233.107	0.975
*CO + $H^+ + e^-$ (TS)	32.867	-0.447	-232.595	0.978

Supplementary Table 6 Fitted parameters for potential-dependent total energies of initial and transition state during the CO_2RR on the Cu(100) surface.

	C (μF/cm^2)	U_0 (V)	E_0 (eV)	R^2
$CO_2 + H^+ + e^-$ (IS)	34.477	-0.307	-199.561	0.980
$CO_2 + H^+ + e^-$ (TS)	32.634	-0.346	-199.331	0.984
*COOH + $H^+ + e^-$ (IS)	32.482	-0.655	-244.196	0.976
*COOH + $H^+ + e^-$ (TS)	33.425	-0.486	-244.000	0.961
*CO + $H^+ + e^-$ (IS)	25.399	-0.478	-236.515	0.973
*CO + $H^+ + e^-$ (TS)	30.871	-0.265	-235.870	0.982

Supplementary Table 7 Fitted parameters for potential-dependent total energies of initial and transition state for the first C-N coupling step on the Cu(111) surface.

	C (μF/cm^2)	U_0 (V)	E_0 (eV)	R^2
CO_2 + *NH (IS)	29.461	-0.164	-213.047	0.985
CO_2 + *NH (TS)	27.400	-0.213	-212.761	0.993
*CO + *NH (IS)	30.218	-0.095	-205.722	0.988
*CO + *NH (TS)	29.003	-0.152	-205.240	0.992

Supplementary Table 8 Fitted parameters for potential-dependent total energies of initial and transition state for the first C-N coupling step on the Cu(110) surface.

	C (μF/cm^2)	U_0 (V)	E_0 (eV)	R^2
CO_2 + *N (IS)	34.723	-0.459	-200.035	0.993
CO_2 + *N (TS)	34.493	-0.465	-199.753	0.993

Supplementary Table 9 Fitted parameters for potential-dependent total energies of initial and transition state for the first C-N coupling step on the Cu(100) surface.

	C (μF/cm^2)	U_0 (V)	E_0 (eV)	R^2
CO_2 + *N (IS)	33.379	-0.357	-203.945	0.983
CO_2 + *N (TS)	31.099	-0.208	-203.490	0.974
*CO + *N (IS)	28.302	-0.320	-196.577	0.992
*CO + *N (TS)	31.479	-0.260	-196.211	0.992
CO_2 + *NH (IS)	31.905	-0.378	-208.531	0.990
CO_2 + *NH (TS)	29.914	-0.254	-207.713	0.990
*COOH + *NH (IS)	27.649	-0.368	-211.922	0.993
*COOH + *NH (TS)	26.509	-0.342	-211.522	0.988
*CO + *NH (IS)	30.932	-0.343	-201.049	0.994
*CO + *NH (TS)	33.182	-0.382	-200.606	0.992

Supplementary Table 10 Fitted parameters for potential-dependent total energies of initial and transition state for hydrogenation steps for *CO_2-N on the Cu(100) surface.

	C (μF/cm^2)	U_0 (V)	E_0 (eV)	R^2
*CO_2-N + H^+ + e^- (IS)	29.351	-0.397	-252.194	0.995
*CO_2-N + H^+ + e^- (TS)	29.336	-0.406	-251.986	0.994
*COOH-N + H^+ + e^- (IS)	31.555	-0.433	-256.727	0.978
*COOH-N + H^+ + e^- (TS)	32.011	-0.403	-256.501	0.976
*CO-N + H^+ + e^- (IS)	32.513	-0.459	-245.691	0.964
*CO-N + H^+ + e^- (TS)	32.634	-0.432	-245.588	0.963

Supplementary Table 11 Fitted parameters for potential-dependent total energies of initial and transition state for the second C-N coupling step and the final hydrogenation steps on the Cu(111) surface.

	C (μF/cm^2)	U_0 (V)	E_0 (eV)	R^2
*CO-NH + *NH(IS)	29.727	-0.115	-217.734	0.994
*CO-NH + *NH(TS)	29.654	-0.118	-217.376	0.995
*NH-CO-NH + H^+ + e^- (IS)	28.844	0.390	-222.234	0.983
*NH-CO-NH + H^+ + e^- (TS)	29.478	0.338	-222.540	0.985
*NH-CO-NH_2 + H^+ + e^- (IS)	28.033	-0.439	-271.275	0.982
*NH-CO-NH_2 + H^+ + e^- (TS)	26.201	-0.476	-270.997	0.986

Supplementary Table 12 Fitted parameters for potential-dependent total energies of initial and transition state for the second C-N coupling step and the final hydrogenation steps on the Cu(100) surface.

	C (μF/cm^2)	U_0 (V)	E_0 (eV)	R^2
*CO-NH + *NH(IS)	31.814	-0.313	-213.401	0.990
*CO-NH + *NH(TS)	33.045	-0.341	-212.926	0.978
*NH-CO-NH + H^+ + e^- (IS)	29.260	-0.474	-262.940	0.990
*NH-CO-NH + H^+ + e^- (TS)	30.567	-0.352	-262.586	0.993
*NH-CO-NH_2 + H^+ + e^- (IS)	30.947	-0.564	-266.273	0.976
*NH-CO-NH_2 + H^+ + e^- (TS)	30.932	-0.576	-266.219	0.974

Supplementary Table 13 Microkinetic equations of urea synthesis process on the Cu(111) surface. * denotes active site. θ_i and P_i represent the coverage and pressure of reactants respectively. C_i is the concentrations of aqueous-phase species.

Reaction steps	Reaction rate (r)
NO_2^- + * = *NO_2 + e^-	$\theta(*NO_2) = K_1 C_{NO_2^-}\theta(*)$
*NO_2 + H^+ + e^- = *HNO_2	$r_2 = k_2\theta(*NO_2)C_{H^+} - k_{-2}\theta(*HNO_2)$
*HNO_2 + H^+ + e^- = *NO + H_2O	$r_3 = k_3\theta(*HNO_2)C_{H^+} - k_{-3}\theta(*NO)C_{H2O}$
*NO + H^+ + e^- =*NOH	$r_4 = k_4\theta(*NO)C_{H^+} - k_{-4}\theta(*NOH)$
*NOH + H^+ + e^- = *N + H_2O	$r_5 = k_5\theta(*NOH)C_{H^+} - k_{-5}\theta(*N)C_{H2O}$
*N + H^+ + e^- = *NH	$r_6 = k_6\theta(*N)C_{H^+} - k_{-6}\theta(*NH)$
CO_2 + * + H^+ + e^- = *COOH	$r_7 = k_7\theta(*)C_{H^+}P_{CO2} - k_{-7}\theta(*COOH)$
*COOH + H^+ + e^- = *CO + H_2O	$r_8 = k_8\theta(*COOH)C_{H^+} - k_{-8}\theta(*CO)C_{H2O}$
*NH + *CO = *CO-NH + * (RDS)	$r_9 = k_9\theta(*NH)\theta(*CO) - k_{-9}\theta(*CO\text{-}NH)\theta(*)$
*CO-NH + *NH = *NH-CO-NH + *	$r_{10} = k_{10}\theta(*CO\text{-}NH)\theta(*NH) - k_{-10}\theta(*NH\text{-}CO\text{-}NH)\theta(*)$
*NH-CO-NH + *H = *NH-CO-NH_2 + *	$r_{11} = k_{11}\theta(*NH\text{-}CO\text{-}NH)\theta(*H) - k_{-11}\theta(*NH\text{-}CO\text{-}NH_2)\theta(*)$
*NH-CO-NH_2 + H^+ + e^- = $CO(NH_2)_2$ + *	$r_{12} = k_{12}\theta(*NH\text{-}CO\text{-}NH_2)C_{H^+} - k_{-12}C_{CO(NH2)2}\theta(*)$
H^+ + e^- + * = *H	$\theta(*H) = K_{13}C_{H^+}\theta(*)$

Supplementary Table 14 Degrees for rate control (X_i) of all intermediates and transition states of urea synthesis process on the Cu(111) surface at 300 K under the applied electrode potential of -1.5 V.

Species (intermediates)	X_i	Species (transition-states)	X_i
$*NO_2$	-1	$*-NO_2$	0
$*HNO_2$	-1	NO_2-H	0
*NO	-1	NOOH-H	0
*NOH	-1	NO-H	0
*N	-1	NOH-H	0
*NH	-1	N-H	0
*COOH	1	COO-H	0
*CO	1	COOH-H	0
*CO-NH	0	CO-NH	1
*NH-CO-NH	0	NHCO-NH	0
$*NH-CO-NH_2$	9.19×10^{-61}	NHCONH-H	0
*	3.15×10^{-60}	$*-CO(NH_2)_2$	0
*H	-3.64×10^{-59}	*-H	0

Supplementary Table 15 Microkinetic equations of urea synthesis process on the Cu(100) surface under the high applied electrode potential (0.00 to -0.42 V). * indicates active site. θ_i and P_i represent the coverage and pressure of reactants respectively. C_i is the concentrations of aqueous-phase species.

$NO_2^- + * = *NO_2 + e^-$	$\theta(*NO_2) = K_1 C_{NO2^-}\theta(*)$
$*NO_2 + H^+ + e^- = *HNO_2$	$r_2 = k_2\theta(*NO_2)C_{H^+} - k_{-2}\theta(*HNO_2)$
$*HNO_2 + H^+ + e^- = *NO + H_2O$	$r_3 = k_3\theta(*HNO_2)C_{H^+} - k_{-3}\theta(*NO)C_{H2O}$
$*NO + H^+ + e^- =*NOH$	$r_4 = k_4\theta(*NO)C_{H^+} - k_{-4}\theta(*NOH)$
$*NOH + H^+ + e^- = *N + H_2O$	$r_5 = k_5\theta(*NOH)C_{H^+} - k_{-5}\theta(*N)C_{H2O}$
*N + *H = *NH + *	$r_6 = k_6\theta(*N)\theta(*H) - k_{-6}\theta(*NH)\theta(*)$
$CO_2 + *H = *COOH$	$r_7 = k_7\theta(*H)P_{CO2} - k_{-7}\theta(*COOH)$
$*COOH + H^+ + e^- = *CO + H_2O$	$r_8 = k_8\theta(*COOH)C_{H^+} - k_{-8}\theta(*CO)C_{H2O}$
*N + *CO = *CO-N + *	$r_9 = k_9\theta(*N)\theta(*CO) - k_{-9}\theta(*CO\text{-}N)\theta(*)$
$*CO-N + H^+ + e^- = *CO-NH$	$r_{10} = k_{10}\theta(*CO\text{-}N)C_{H^+} - k_{-10}\theta(*CO\text{-}NH)$
*CO-NH + *NH = *NH-CO-NH + * (RDS)	$r_{11} = k_{11}\theta(*CO\text{-}NH)\theta(*NH) - k_{-11}\theta(*NH\text{-}CO\text{-}NH)\theta(*)$
$*NH-CO-NH + H^+ + e^- = *NH-CO-NH_2$	$r_{12} = k_{12}\theta(*NH\text{-}CO\text{-}NH)C_{H^+} - k_{-12}\theta(*NH\text{-}CO\text{-}NH_2)$
$*NH-CO-NH_2 + H^+ + e^- = CO(NH_2)_2 + *$	$r_{13} = k_{13}\theta(*NH\text{-}CO\text{-}NH_2)C_{H^+} - k_{-13}C_{CO(NH2)2}\theta(*)$
$H^+ + e^- + * = *H$	$\theta(*H) = K_{14}C_{H^+}\theta(*)$

Supplementary Table 16 Microkinetic equations of urea synthesis process on the Cu(100) surface under the low applied electrode potential (-0.42 to -1.30 V). * indicates active site. θ_i and P_i represent the coverage and pressure of reactants respectively. C_i is the concentrations of aqueous-phase species.

$NO_2^- + * = *NO_2 + e^-$	$\theta(*NO_2) = K_1 C_{NO2^-}\theta(*)$
$*NO_2 + H^+ + e^- = *HNO_2$ (RDS)	$r_2 = k_2\theta(*NO_2)C_{H^+} - k_{-2}\theta(*HNO_2)$
$*HNO_2 + H^+ + e^- = *NO + H_2O$	$r_3 = k_3\theta(*HNO_2)C_{H^+} - k_{-3}\theta(*NO)C_{H2O}$
$*NO + H^+ + e^- =*NOH$	$r_4 = k_4\theta(*NO)C_{H^+} - k_{-4}\theta(*NOH)$
$*NOH + H^+ + e^- = *N + H_2O$	$r_5 = k_5\theta(*NOH)C_{H^+} - k_{-5}\theta(*N)C_{H2O}$
*N + *H = *NH + *	$r_6 = k_6\theta(*N)\theta(*H) - k_{-6}\theta(*NH)\theta(*)$
$*N + CO_2 = *CO_2-N$	$r_7 = k_7\theta(*N)P_{CO2} – k_{-7}\theta(*CO_2\text{-}N)$
$*CO_2-N + H^+ + e^- = *COOH-N$	$r_8 = k_8\theta(*CO_2\text{-}N)C_{H^+} - k_{-8}\theta(*COOH\text{-}N)$
$*COOH-N + H^+ + e^- = *CO-N + H_2O$	$r_9 = k_9\theta(*COOH\text{-}N)C_{H^+} - k_{-9}\theta(*CO\text{-}N)C_{H2O}$
$*CO-N + H^+ + e^- = *CO-NH$	$r_{10} = k_{10}\theta(*CO\text{-}N)C_{H^+} - k_{-10}\theta(*CO\text{-}NH)$

$*CO\text{-}NH + *NH = *NH\text{-}CO\text{-}NH + *$	$r_{11} = k_{11}\theta(*CO\text{-}NH)\theta(*NH) - k_{-11}\theta(*NH\text{-}CO\text{-}NH)\theta(*)$
$*NH\text{-}CO\text{-}NH + H^+ + e^- = *NH\text{-}CO\text{-}NH_2$	$r_{12} = k_{12}\theta(*NH\text{-}CO\text{-}NH)C_{H^+} - k_{-12}\theta(*NH\text{-}CO\text{-}NH_2)$
$*NH\text{-}CO\text{-}NH_2 + H^+ + e^- = CO(NH_2)_2 + *$	$r_{13} = k_{13}\theta(*NH\text{-}CO\text{-}NH_2)C_{H^+} - k_{-13}C_{CO(NH2)2}\theta(*)$
$H^+ + e^- + * = *H$	$\theta(*H) = K_{14}C_{H^+}\theta(*)$

Supplementary Table 17 Degrees for rate control (X_i) of all intermediates and transition states of urea synthesis process on the Cu(100) surface at 300 K under the applied electrode potential of -0.5 V.

Species (intermediates)	X_i	Species (transition-states)	X_i
$*NO_2$	2.17×10^{-23}	$*\text{-}NO_2$	0
$*HNO_2$	0	$NO_2\text{-}H$	1
*NO	0	NOOH-H	0
*NOH	0	NO-H	0
*N	0	NOH-H	0
*NH	3.12×10^{-32}	N-H	0
$*CO_2\text{-}N$	-3.12×10^{-32}	$CO_2\text{-}N$	0
*COOH-N	-3.12×10^{-32}	NCOO-H	0
*CO-N	-3.12×10^{-32}	CO-N	0
*CO-NH	-3.12×10^{-32}	CON-H	0
*NH-CO-NH	3.12×10^{-32}	NHCO-NH	0
$*NH\text{-}CO\text{-}NH_2$	2.33×10^{-27}	NHCONH-H	0
*	2.33×10^{-27}	$*\text{-}CO(NH_2)_2$	0
*H	-1.02×10^{-23}	*-H	0

Supplementary Table 18 Microkinetic equations of NO_2^-RR process on Cu(111) surface. * indicates active site. θ_i and P_i represent the coverage and pressure of reactants respectively. C_i is the concentrations of aqueous-phase species.

$NO_2^- + * = *NO_2 + e^-$	$\theta(*NO_2) = K_1C_{NO2^-}\theta(*)$
$*NO_2 + H^+ + e^- = *HNO_2$	$r_2 = k_2\theta(*NO_2)C_{H^+} - k_{-2}\theta(*HNO_2)$
$*HNO_2 + H^+ + e^- = *NO + H_2O$	$r_3 = k_3\theta(*HNO_2)C_{H^+} - k_{-3}\theta(*NO)C_{H2O}$
$*NO + H^+ + e^- =*NOH$	$r_4 = k_4\theta(*NO)C_{H^+} - k_{-4}\theta(*NOH)$
$*NOH + H^+ + e^- = *N + H_2O$	$r_5 = k_5\theta(*NOH)C_{H^+} - k_{-5}\theta(*N)C_{H2O}$
$*N + H^+ + e^- = *NH$	$r_6 = k_6\theta(*N)C_{H^+} - k_{-6}\theta(*NH)$
$*NH + *H = *NH_2 + *$ (RDS)	$r_7 = k_7\theta(*NH)\theta(*H) - k_{-7}\theta(*NH_2)\theta(*)$
$*NH_2 + H^+ + e^- = * + NH_3$	$r_8 = k_8\theta(*NH_2)C_{H^+} - k_{-8}P_{NH3}\theta(*)$
$H^+ + e^- + * = *H$	$\theta(*H) = K_9C_{H^+}\theta(*)$

Supplementary Table 19 Degrees for rate control (X_i) of all intermediates and transition states of NO^{2-}RR process on the Cu(111) surface at 300 K under the applied electrode potential of -1.5 V.

Species (intermediates)	X_i	Species (transition-states)	X_i
$*NO_2$	-1	$*\text{-}NO_2$	0
$*HNO_2$	-1	$NO_2\text{-}H$	0
*NO	-1	NOOH-H	0
*NOH	-1	NO-H	0
*N	-1	NOH-H	0
*NH	-1	N-H	0
$*NH_2$	2.96×10^{-71}	NH-H	1
*	2.96×10^{-71}	$*\text{-}NH_3$	0

*H	2.96×10^{-71}	*-H	0

Supplementary Table 20 Microkinetic equations of NO_2^-RR process on Cu(100) surface. * indicates active site. θ_i and P_i represent the coverage and pressure of reactants respectively. C_i is the concentrations of aqueous-phase species.

$NO_2^- + * = *NO_2 + e^-$	$\theta(*NO_2) = K_1C_{NO2^-}\theta(*)$
$*NO_2 + H^+ + e^- = *HNO_2$	$r_2 = k_2\theta(*NO_2)C_{H^+} - k_{-2}\theta(*HNO_2)$
$*HNO_2 + H^+ + e^- = *NO + H_2O$	$r_3 = k_3\theta(*HNO_2)C_{H^+} - k_{-3}\theta(*NO)C_{H2O}$
$*NO + H^+ + e^- = *NOH$	$r_4 = k_4\theta(*NO)C_{H^+} - k_{-4}\theta(*NOH)$
$*NOH + H^+ + e^- = *N + H_2O$	$r_5 = k_5\theta(*NOH)C_{H^+} - k_{-5}\theta(*N)C_{H2O}$
$*N + *H = *NH + *$	$r_6 = k_6\theta(*N)\theta(*H) - k_{-6}\theta(*NH)\theta(*)$
$*NH + H^+ + e^- = *NH_2$ (RDS)	$r_7 = k_7\theta(*NH)C_{H^+} - k_{-7}\theta(*NH_2)$
$*NH_2 + H^+ + e^- = * + NH_3$	$r_8 = k_8\theta(*NH_2)C_{H^+} - k_{-8}P_{NH3}\theta(*)$
$H^+ + e^- + * = *H$	$\theta(*H) = K_9C_{H^+}\theta(*)$

Supplementary Table 21 Degrees for rate control (X_i) of all intermediates and transition states of NO^{2-}RR process on the Cu(100) surface at 300 K under the applied electrode potential of -0.75 V.

Species (intermediates)	X_i	Species (transition-states)	X_i
$*NO_2$	0	$*-NO_2$	0
$*HNO_2$	5.97×10^{-72}	NO_2-H	0
*NO	5.97×10^{-72}	NOOH-H	0
*NOH	1.59×10^{-50}	NO-H	0
*N	1.25×10^{-42}	NOH-H	0
*NH	6.24×10^{-18}	N-H	0
$*NH_2$	0	NH-H	1
*	0	$*-NH_3$	0
*H	6.24×10^{-18}	*-H	0

Supplementary Table 22 Microkinetic equations of CO_2RR process on Cu(111) surface. * indicates active site. θ_i and P_i represent the coverage and pressure of reactants respectively. C_i is the concentrations of aqueous-phase species.

$CO_2 + * = *CO_2$	$\theta(*CO_2) = K_1P_{CO2}\theta(*)$
$*CO_2 + H^+ + e^- = *COOH$	$r_2 = k_2\theta(*CO_2)C_{H^+} - k_{-2}\theta(*COOH)$
$*COOH + H^+ + e^- = *CO + H_2O$	$r_3 = k_3\theta(*COOH)C_{H^+} - k_{-3}\theta(*CO)C_{H2O}$
$*CO = * + CO$ (RDS)	$r_4 = k_4\theta(*CO) - k_{-4}\theta(*)P_{CO}$

Supplementary Table 23 Microkinetic equations of CO_2RR process on Cu(100) surface. * indicates active site. θ_i and P_i represent the coverage and pressure of reactants respectively. C_i is the concentrations of aqueous-phase species.

$CO_2 + * = *CO_2$	$\theta(*CO_2) = K_1P_{CO2}\theta(*)$
$*CO_2 + *H = *COOH + *$	$r_2 = k_2\theta(*CO_2)\theta(*H) - k_{-2}\theta(*COOH)\theta(*)$
$*COOH + H^+ + e^- = *CO + H_2O$	$r_3 = k_3\theta(*COOH)C_{H^+} - k_{-3}\theta(*CO)C_{H2O}$
$*CO = * + CO$ (RDS)	$r_4 = k_4\theta(*CO) - k_{-4}\theta(*)P_{CO}$
$H^+ + e^- + * = *H$	$\theta(*H) = K_5C_{H^+}\theta(*)$

Supplementary Table 24 Calculated Gibbs free energy values (ΔG) for reaction steps during the NO_2^-RR on Cu(111), Cu(110) and Cu(100) surfaces without extra charges added, corresponding to

Supplementary Table 18. The unit is eV.

Cu(111)	$*NO_2$	$*HNO_2$	*NO	*NOH	*N	*NH	$*NH_2$	$*NH_3$
Δ*G* (eV) (L-H)	-0.929	0.315	-1.552	0.145	-0.968	-1.131	-0.461	-0.543
Δ*G* (eV) (E-R)	-1.567	0.554	-1.864	0.112	-1.010	-0.951	-0.385	-0.951
Cu(110)	$*NO_2$	$*HNO_2$	*NO	*NOH	*N	*NH	$*NH_2$	$*NH_3$
Δ*G* (eV) (L-H)	-1.992	0.688	-1.401	0.350	-1.556	-0.640	-0.987	-0.381
Δ*G* (eV) (E-R)	-1.086	0.806	-1.138	0.224	-1.495	-0.716	-1.046	-1.039
Cu(100)	$*NO_2$	$*HNO_2$	*NO	*NOH	*N	*NH	$*NH_2$	$*NH_3$
Δ*G* (eV) (L-H)	-1.197	0.548	-1.265	-0.344	-1.423	-0.857	-0.246	-0.484
Δ*G* (eV) (E-R)	-1.879	0.705	-1.357	-0.440	-1.211	-0.767	-0.315	-0.737

Supplementary Table 25 Calculated Gibbs free energy values (Δ*G*) for reaction steps during the CO_2RR on Cu(111), Cu(110) and Cu(100) surfaces without extra charges added, corresponding to Supplementary Table 19. The unit is eV.

Cu(111)	$*CO_2$	*COOH	*CO	*HCO	CO (g)
Δ*G* (eV) (L-H)	-0.037	0.383	-0.372	0.882	1.022
Δ*G* (eV) (E-R)	0.414	0.382	-0.270	0.660	
Cu(110)	$*CO_2$	*COOH	*CO	*HCO	CO (g)
Δ*G* (eV) (L-H)	0.089	0.118	-0.447	0.931	1.342
Δ*G* (eV) (E-R)	0.653	0.024	-0.179	-0.474	
Cu(100)	$*CO_2$	*COOH	*CO	*HCO	CO (g)
Δ*G* (eV) (L-H)	0.028	0.279	-0.376	0.668	1.069
Δ*G* (eV) (E-R)	0.566	0.115	-0.256	-0.108	

Supplementary Table 26 Calculated Gibbs free energy values (Δ*G*) for the possible coupling steps (the first coupling step) during the urea synthesis on Cu(111), Cu(110) and Cu(100) surfaces without extra charges added. The unit is eV.

Cu(111)			*NO	*NOH	*N	*NH	
Δ*G* (eV) ($*CO_2$)			0.447	-0.341	-0.450	-0.362	
Δ*G* (eV) (*CO)			0.744	0.040	-1.591	0.019	
Cu(110)	$*NO_2$	$*HNO_2$	*NO	*NOH	*N	*NH	$*NH_2$
Δ*G* (eV) ($*CO_2$)	-0.391	-0.601	-0.599	-1.461	-1.134	-1.106	-0.570
Δ*G* (eV) (*CO)			0.427	-0.836	-2.060	-0.678	0.134
Cu(100)	$*NO_2$	$*HNO_2$	*NO	*NOH	*N	*NH	
Δ*G* (eV) ($*CO_2$)	-0.211	-0.217	-0.003	-0.681	0.035	-0.006	
Δ*G* (eV) (*COOH)	-0.471	-0.131	-1.123	-0.962	-1.057	-1.157	
Δ*G* (eV) (*CO)			0.138	-0.402	-1.021	-0.213	

Supplementary Table 27 Calculated Gibbs free energy values (Δ*G*) for the possible coupling steps (the second coupling step) during the urea synthesis on Cu(111) and Cu(100) surfaces without extra charges added. The unit is eV.

Cu(111)	*NH-CO-NOH	*NH-CO-NH
Δ*G*	-0.512	-1.250

Cu(100)	*N-COOH-N	*N-CO-NH	*NH-CO-NH
Δ*G*	-1.248	-0.948	-1.413

Supplementary Table 28 Calculated Gibbs free energy values (ΔG) for reaction steps during the urea synthesis on Cu(111) and Cu(100) surfaces with respect to the applied electrode potentials (vs RHE), corresponding to Supplementary Tables 13,15,16. The unit is eV.

Cu(111)	-1.50 V	-1.25 V	-1.00 V	-0.75 V	-0.50 V	-0.25 V	0.00 V
ΔG(*NO$_2$)	-1.113	-1.152	-1.192	-1.232	-1.257	-1.204	-1.131
ΔG(*NO$_2$H)	0.351	0.311	0.271	0.228	0.182	0.132	0.079
ΔG(*NO)	-2.532	-2.546	-2.560	-2.529	-2.436	-2.331	-2.215
ΔG(*NOH)	-0.304	-0.298	-0.292	-0.283	-0.230	-0.171	-0.116
ΔG(*N)	-1.463	-1.501	-1.538	-1.551	-1.560	-1.569	-1.578
ΔG(*NH)	-1.315	-1.332	-1.349	-1.350	-1.337	-1.321	-1.300
ΔG(*COOH)	0.064	0.004	-0.055	-0.070	-0.069	-0.058	-0.037
ΔG(*CO)	-0.896	-0.902	-0.908	-0.919	-0.937	-0.961	-0.990
ΔG(*CO-NH)	0.041	0.024	0.008	-0.009	-0.012	-0.004	0.005
ΔG(*NH-CO-NH)	-1.212	-1.228	-1.243	-1.258	-1.264	-1.262	-1.258
ΔG(*NH-CO-NH$_2$)	-0.227	-0.245	-0.264	-0.288	-0.381	-0.493	-0.601
ΔG(*NH$_2$-CO-NH$_2$)	-0.533	-0.535	-0.541	-0.551	-0.556	-0.554	-0.547

Cu(100)	-1.50 V	-1.25 V	-1.00 V	-0.75 V	-0.50 V	-0.25 V	0.00 V
ΔG(*NO$_2$)	-1.171	-1.232	-1.293	-1.354	-1.398	-1.363	-1.302
ΔG(*NO$_2$H)	0.539	0.476	0.414	0.312	0.198	0.103	0.026
ΔG(*NO)	-2.252	-2.333	-2.414	-2.435	-2.393	-2.321	-2.220
ΔG(*NOH)	-0.911	-0.897	-0.884	-0.867	-0.829	-0.801	-0.783
ΔG(*N)	-1.828	-1.846	-1.865	-1.866	-1.802	-1.736	-1.672
ΔG(*NH)	-0.919	-0.916	-0.913	-0.910	-0.918	-0.935	-0.957
ΔG(*CO$_2$-N)	0.092	0.036	-0.021	-0.078	-0.104		
ΔG(*COOH-N)	-1.339	-1.321	-1.302	-1.283	-1.262		
ΔG(*COOH)						0.179	0.138
ΔG(*CO)						-0.986	-0.993
ΔG(*CO-N)	-1.237	-1.215	-1.192	-1.169	-1.113	-1.058	-1.026
ΔG(*CO-NH)	-0.720	-0.773	-0.826	-0.888	-0.932	-0.951	-0.945
ΔG(*NH-CO-NH)	-1.470	-1.475	-1.480	-1.485	-1.471	-1.447	-1.424
ΔG(*NH-CO-NH$_2$)	-0.154	-0.179	-0.205	-0.242	-0.274	-0.299	-0.319
ΔG(*NH$_2$-CO-NH$_2$)	-0.578	-0.606	-0.635	-0.668	-0.696	-0.720	-0.740

For urea synthesis on Cu(111) and Cu(100) surfaces, we have identified significant impacts from various intermediates and transition states on turnover frequency (TOF), as summarized in Supplementary Table 14. For the Cu(111) surface, N-intermediates such as *NO$_2$, *HNO$_2$, *NO, *NOH, *N, and *NH adversely affect the reaction rate, while C-intermediates like *COOH, *CO, and the transition state of *CO-NH positively influence it. The behaviors of these intermediates and transition sate under various applied electrode potentials are detailed in Supplementary Table 28 and Fig. 2 in the manuscript. Notably, intermediates *NO$_2$, *HNO$_2$, *N, *COOH, *CO, and the transition state *CO-NH become increasingly destabilized with more negative electrode potentials. In contrast, intermediates *NO and *NOH stabilize under such conditions. The most significant variation is observed in the free energy of *NO$_2$, which alters by approximately 1 eV across applied electrode potential range of 0 to -1.5 V. In comparison, the changes in other intermediates and the transition state *CO-NH are less pronounced, remaining below 0.3 eV. These behaviors correlate directly with the observed TOF trends, where a more negative electrode potential leads to an increase in TOF, primarily due to the rising free energy of *NO$_2$ during urea synthesis on the Cu(111) surface. The

relationship is also consistent with the TOF trends during the NO_2^-RR on the Cu(111) surface, as demonstrated in Supplementary Tables 18,19,28.

On the Cu(100) surface, the TOF values are significantly influenced by intermediates *NO_2, *H, and the transition state *NO_2-H. Here, intermediate *NO_2 and the transition state *NO_2-H positively impact the reaction rate, while the *H intermediate has a negative effect. As shown in Supplementary Table 28 and Supplementary Fig.12, both the intermediate *NO_2 and the transition state *NO_2-H become more destabilized as the electrode potential becomes more negative, while the intermediate *H stabilizes under these conditions. This dynamic between stabilization and destabilization leads to an increase in TOF as the electrode potential being more negative, a trend consistent with the NO_2^-RR on the Cu(100) surface, as illustrated in Fig. 4 in the manuscript.

Supplementary Table 29 Calculated energy, free energy correction and corresponding Gibbs free energy values for NH_3, CO_2, CO, H_2O and H_2. The unit is eV.

	E (eV)	*ZPE-TS* (eV)	*G* (eV)
NH_3	-19.542	0.327	-19.215
CO_2	-22.952	-0.349	-23.301
CO	-14.780	-0.468	-15.248
H_2O	-14.220	-0.110	-14.330
H_2	-6.780	-0.120	-6.900

Noted: The energy of a proton and electron pair ($H^+ + e^-$) is calculated as: $G(H^+ + e^-) = 1/2\ G(H_2)$.

Supplementary References

1. Beinlich, S. D., Hörmann, N. G., & Reuter, K. Field effects at protruding defect sites in electrocatalysis at metal electrodes? *ACS Catal.* 12, 6143–6148 (2022).
2. Akhade, S. A., Bernstein, N. J., Esopi, M. R., Regula, M. J., & Janik, M. J. A simple method to approximate electrode potential-dependent activation energies using density functional theory. *Catal. Today* **288**, 63–73 (2017).
3. Tran, B., Cai, Y., Janik, M. J., & Milner, S. T. Hydrogen bond thermodynamics in aqueous acid solutions: a combined DFT and classical force-field approach. *J. Phys. Chem. A* **126**, 7382–7398 (2022).
4. Arntsen, C., Chen, C., Calio, P. B., Li, C., & Voth, G. A. The hopping mechanism of the hydrated excess proton and its contribution to proton diffusion in water. *J. Chem. Phys.* **154** (2021).
5. Calio, P. B., Li, C., & Voth, G. A. Resolving the structural debate for the hydrated excess proton in water. *J. Am. Chem. Soc.* **143**, 18672–18683 (2021).
